# Phosphodiesterase Inhibitors in Acute Lung Injury: What Are the Perspectives?

**DOI:** 10.3390/ijms22041929

**Published:** 2021-02-16

**Authors:** Daniela Mokra, Juraj Mokry

**Affiliations:** 1 Department of Physiology, Jessenius Faculty of Medicine in Martin, Comenius University in Bratislava, 03601 Martin, Slovakia; 2 Department of Pharmacology, Jessenius Faculty of Medicine in Martin, Comenius University in Bratislava, 03601 Martin, Slovakia; juraj.mokry@uniba.sk

**Keywords:** acute lung injury, acute respiratory distress syndrome, sepsis, phosphodiesterase inhibitors, animal models, COVID-19

## Abstract

Despite progress in understanding the pathophysiology of acute lung damage, currently approved treatment possibilities are limited to lung-protective ventilation, prone positioning, and supportive interventions. Various pharmacological approaches have also been tested, with neuromuscular blockers and corticosteroids considered as the most promising. However, inhibitors of phosphodiesterases (PDEs) also exert a broad spectrum of favorable effects potentially beneficial in acute lung damage. This article reviews pharmacological action and therapeutical potential of nonselective and selective PDE inhibitors and summarizes the results from available studies focused on the use of PDE inhibitors in animal models and clinical studies, including their adverse effects. The data suggest that xanthines as representatives of nonselective PDE inhibitors may reduce acute lung damage, and decrease mortality and length of hospital stay. Various (selective) PDE3, PDE4, and PDE5 inhibitors have also demonstrated stabilization of the pulmonary epithelial–endothelial barrier and reduction the sepsis- and inflammation-increased microvascular permeability, and suppression of the production of inflammatory mediators, which finally resulted in improved oxygenation and ventilatory parameters. However, the current lack of sufficient clinical evidence limits their recommendation for a broader use. A separate chapter focuses on involvement of cyclic adenosine monophosphate (cAMP) and PDE-related changes in its metabolism in association with coronavirus disease 2019 (COVID-19). The chapter illuminates perspectives of the use of PDE inhibitors as an add-on treatment based on actual experimental and clinical trials with preliminary data suggesting their potential benefit.

## 1. Introduction

An acute lung damage originating from a variety of direct (pulmonary) or indirect (extrapulmonary) reasons is characterized by an acute development of respiratory distress and hypoxemia resulting from diffuse damage of an alveolar–capillary barrier, dysfunction of a pulmonary surfactant, massive generation of a lung edema, and ventilation-perfusion mismatch, followed by neutrophil-mediated inflammation. Despite substantial improvement in understanding the pathophysiology of this life-threatening situation, treatment possibilities are limited to lung-protective ventilation, prone positioning, and supportive interventions. Various pharmacological approaches have been also tested, with neuromuscular blockers and corticosteroids considered as the most promising. However, there are additional drugs, e.g., inhibitors of phosphodiesterases (PDEs), which should be mentioned because of their wide spectrum of potentially favorable effects. This article reviews pharmacological properties of nonselective and selective PDE inhibitors, their mechanisms of action, side effects, and therapeutical potential for the acutely injured lung, summarizes results from the use of PDE inhibitors in the experimental and clinical studies, and estimates possible perspectives of a wider use of PDE inhibitors for an acute lung damage including coronavirus disease 2019 (COVID-19).

## 2. Acute Injury of the Lung

### 2.1. Definitions

The American–European Consensus Conference definitions state the following as the main features of acute lung damage: (1) an acute onset; (2) finding of bilateral infiltrates on the chest X-ray; (3) pulmonary artery wedge pressure ≤ 18 mm Hg or an absence of a clinical evidence of left atrial hypertension; and (4) an acute hypoxemia. The term acute lung injury (ALI) represents a situation with a ratio of arterial partial pressure of oxygen and fraction of inspired oxygen (PaO_2_/FiO_2_) > 200 mm Hg and < 300 mm Hg. The term acute respiratory distress syndrome (ARDS) is used for more severe situations with the PaO_2_/FiO_2_ ≤ 200 mm Hg [1].

The so-called Berlin definition of ARDS in 2012 defined “ARDS” as an acute form of diffuse lung injury occurring in patients with a predisposing risk factor, meeting the following criteria: (1) an onset within 1 week of a known clinical insult or new/worsening respiratory symptoms; (2) presence of bilateral opacities on the chest X-ray, not fully explained by effusion, lobar/lung collapse, or nodules; (3) diagnosis of respiratory failure not fully explained by cardiac failure or fluid overload; (4) presence of hypoxemia, as defined by a specific threshold of the PaO_2_/FiO_2_ ratio measured with a minimum requirement of positive end-expiratory pressure (PEEP) ≥ 5 cm H_2_O; thus, identifying three categories of ARDS based on a severity of hypoxemia: mild (PaO_2_/FiO_2_ 200–300 mm Hg), moderate (PaO_2_/FiO_2_ 100–200 mm Hg), and severe ARDS (PaO_2_/FiO_2_ ≤100 mm Hg) [2]. The term “acute lung injury” has remained as a general term expressing acute damage of the lung, as well as for experimental studies, where the lung damage is induced artificially, and other signs from the definition, except of hypoxemia, cannot be measured.

### 2.2. Incidence and Mortality

Incidence of ARDS may differ according to regional genetic variability, differences in health care systems, and different diagnostic criteria for ARDS [3]. In 2005, incidence of mild ARDS was 79 cases per 100,000; for moderate/severe ARDS, it was 59 cases per 100,000, with a total mortality of about 40% [4]. Later, a declining trend of the ARDS incidence was demonstrated, probably thanks to an introduction of the lung-protective ventilation. In a multicenter trial executed in 50 countries in five continents, ARDS caused 10.4% of total intensive care unit (ICU) admissions and 23.4% of all cases requiring lung ventilation. The prevalence of mild ARDS was 30.0%, of moderate ARDS 46.6%, and of severe ARDS 23.4%, while overall, unadjusted ICU and hospital mortality was 35.3 and 40.0%, respectively and both raised with worsening ARDS severity [5]. Moreover, ARDS is responsible for serious long-term consequences in the patients who had survived ARDS, in terms of a reduced quality of life and physical and mental dysfunction, to a various extent [6,7].

### 2.3. Etiology and Risk Factors

An acute lung damage can be caused by a direct (pulmonary) injury due to pneumonia, near-drowning, inhalation of gases, etc., while an indirect (extrapulmonary) injury is a secondary hit of a systemic process or a serious disorder localized in other organs, such as sepsis, acute pancreatitis, trauma with shock, burns, etc. [8]. However, additional factors may increase a risk of ARDS. For instance, genetic factors may predispose an individual susceptibility for a respiratory distress. More than 40 candidate genes associated with a development of ARDS [9] have been identified, including genes for angiotensin-converting enzyme (ACE), interleukins (IL)-6, and IL-10, tumor necrosis factor (TNF)α, surfactant protein (SP)-B, Fas, vascular endothelial growth factor (VEGF), etc., which are responsible for regulation of vascular permeability, immune response, response to oxidative stress, coagulation, and metabolism in the lung [10,11]. Higher risk of ARDS or a more severe course of the disorder might be partially attributable to race differences [12], blood type [13], chronic cigarette smoke exposure, chronic alcohol abuse, older age (>65 years), chronic lung disease, serious concomitant diseases, etc. [14,15].

### 2.4. Pathophysiological Features of ALI/ARDS

The fundamental changes in ALI/ARDS include increased permeability of the alveolar-capillary membrane, excessive accumulation and activation of leukocytes and platelets, neutrophil-mediated inflammation, and activation of the coagulation pathways [14]. The changes in ALI/ARDS fluently progress from an initial (exudative) phase into a fibroproliferative phase. In the exudative phase (day 1–7), a diffuse injury to epithelial and/or endothelial cells causes a swamping of the alveoli with a proteinaceous edema fluid, which dilutes and deteriorates functions of the pulmonary surfactant. The mentioned processes decrease lung compliance and worsen gas exchange. The lung tissue damage triggers the inflammatory response with a transmigration of leukocytes, particularly neutrophils, from the blood flow into the lung. The immune cells, but also platelets, epithelial, and endothelial cells, and other cells localized at a site of injury, activate and generate a wide spectrum of biologically active substances, such as pro-inflammatory cytokines and chemokines (TNFα, interleukins (IL)-1β, -6, and -8, proteases, reactive oxygen species (ROS), etc. The substances produced by the activated cells or released from the destroyed cells stimulate a chemotaxis of additional immune cells into the lung, which aggravate the injury, but might be also used as biomarkers of the acute phase of ALI/ARDS [16,17]. Several pro-inflammatory mediators induce an apoptosis of the lung epithelial cells, which further disrupt an alveolar lining, while they postpone an apoptosis of neutrophils, allowing their longer persistence and production of deleterious products in the tissue [18]. Within several days, the inflammation becomes weaker and the fibroproliferative processes dominate. The edema fluid is gradually reabsorbed and a damaged tissue goes through a regeneration by proliferation and phenotypic changes of type II alveolar cells, myofibroblasts, and fibroblasts, and a new extracellular matrix becomes deposited. In alteration of the recovery phase, the situation in some patients may progress into a fibrosis and irreversible changes in the lung architecture [17,19].

### 2.5. Therapy of ARDS

Due to a complex pathophysiology and a heterogenous picture of ARDS, the therapy is extremely difficult. The standard therapy is based on a lung-protective mechanical ventilation, which uses low tidal volumes (<6 mL/kg of the predicted body weight) combined with limited inspiratory plateau pressures (< 30 cm H_2_O) and appropriate levels of PEEP [20,21]. To re-inflate the collapsed lung regions, recruitment maneuvers (sustained inflation, intermittent sighs, or stepwise increase in an inspiratory pressure) might be used; however, their benefit is still discussed [22]. Further advances were reported for prone positioning, which enables a recruitment of the lung parenchyma in the acute phase of severe ARDS [20,23]. The improvement in a ventilation/perfusion matching leads to a better CO_2_ elimination and a more homogenous distribution of ventilation [24].

In the pharmacological therapy of ARDS, various medicaments have been used; however, only some of them were able to improve oxygenation, or reduce mortality or duration of hospital stay—at least in the specific groups of patients [25,26]. Among them, neuromuscular blockers and corticosteroids appear to be the most promising. Neuromuscular blockers, thanks to the amelioration of a patient–ventilator synchrony, may reduce the work of breathing and oxygen consumption and, thereby, improve survival [24,27]. Low-to-moderate doses of corticosteroids may shorten a duration of mechanical ventilation or a duration of hospitalization, and improve oxygenation [28,29,30]. Nevertheless, inhibitors of phosphodiesterases (PDEs) represent another option of the pharmacological strategy, exerting a broad spectrum of potentially desired actions, which might be of benefit in ARDS.

## 3. Phosphodiesterases (PDEs)

Intracellular second messengers, a cyclic adenosine monophosphate (cAMP) and a cyclic guanosine monophosphate (cGMP), are involved in the regulation of a variety of physiological processes, including vascular resistance, cardiac output, neuroplasticity, immune response, and inflammation. Intracellular levels of these cyclic nucleotides are regulated particularly by enzymes phosphodiesterases, which catalyze a hydrolysis of a cyclic phosphate bond in the cAMP and cGMP to generate the products 5′-AMP and 5′-GMP, which are inactive.

Thus, PDEs may modulate cell signaling via the breakdown of cAMP and/or cGMP [31,32]. A superfamily of PDEs comprises 11 families of enzymes hydrolyzing cAMP only (PDE4, PDE7, PDE8), cGMP only (PDE5, PDE6, PDE9), or both cAMP and cGMP (PDE1, PDE2, PDE3, PDE10, PDE11). The PDE families may consist of one or more genes, which may give rise to more than 50 protein forms of these enzymes [33]. Distribution of PDEs is diverse in different cells and tissues, while some cells can produce several types of PDEs. Inhibitors of PDEs target one or several PDE isoenzymes, and thereby inhibit a metabolism of the cyclic nucleotides and prolong their biological effects [31,32,34]. The distribution of PDEs, biological effects, and examples of their inhibitors are provided in Table 1.

Because of a fundamental role of the lung epithelial and endothelial cells, neutrophils, macrophages, T-lymphocytes, vascular smooth muscle cells, etc., in the pathogenesis of ALI/ARDS, inhibition of PDE3, PDE4, and PDE5 isoforms distributed in the mentioned cells by either nonselective PDE inhibitors or selective PDE3, PDE4, and PDE5 inhibitors is of a particular interest in ARDS. Nevertheless, inhibitors of other PDEs distributed in the lung (PDE1, PDE2, PDE9) might also be of benefit.

## 4. Nonselective Inhibitors of PDEs

### 4.1. Therapeutic Properties of Nonselective PDE Inhibitors

Nonselective inhibitors of PDEs may modulate simultaneously an action of several PDEs distributed in various tissues; however, this wide-spectrum effect is often associated with unwanted side effects. Nonselective PDE inhibitors are represented by a group of xanthines, since some of them (e.g., theophylline (1,3-dimethylxanthine), caffeine (1,3,7-trimethylxanthine) or theobromine (3,7-dimethylxanthine)) are present as natural alkaloids in tea, coffee, cocoa, etc. [35].

Theophylline has been used in the treatment of bronchial asthma and chronic obstructive pulmonary disease (COPD) for more than 50 years. Actions of theophylline are mediated via several mechanisms while some of them have not been fully understood. Nonselective PDE inhibition and antagonism of adenosine receptors are usually observed at higher doses of theophylline. In addition, theophylline possesses anti-inflammatory properties mediated via increasing activity of histone deacetylases (HDAC), inhibition of phosphoinositide (PI) 3-kinase, inhibition of activity of poly (ADP-ribose) polymerase (PARP-1), or activation of small and intermediate conductance calcium-activated potassium channels [36]. As a result of these actions, administration of theophylline can lead to: (1) bronchodilation and bronchoprotection; (2) vasodilation; (3) anti-inflammatory effects, such as inhibition of nuclear factor (NF)-κB, increased production of anti-inflammatory IL-10, antagonism of TNFα, inhibition of ROS generation, increased apoptosis of neutrophils, inhibition of calcium influx into inflammatory cells, etc.; and (4) other effects, such as facilitation of mucociliary clearance, decrease of edema formation, stimulation of surfactant release, stimulation of breathing etc. [37,38,39].

Aminophylline is a combination of theophylline with ethylenediamine in a 2:1 ratio. Although aminophylline is less potent and shorter-acting than theophylline, the addition of ethylenediamine enhances water-solubility and antioxidant properties compared to theophylline [40].

Pentoxifylline (or oxpentifylline) is widely used for reduction of pain and weakness in peripheral artery diseases [41] and in alcoholic hepatitis, reducing a risk of hepatorenal syndrome [42]. These effects are probably attributable to its anti-inflammatory action including a downregulation of TNFα and anti-thrombotic effects [43].

Lisofylline is an active metabolite of pentoxifylline, possessing similar anti-inflammatory effects. In addition, lisofylline inhibits a formation of oleate- and linoleate-containing phosphatidic acids by neutrophils, and may thereby reduce the oxidant-mediated vascular leak [44].

Caffeine is known for its psychostimulating effects. However, because of stimulation of respiration, caffeine is also used for the treatment of apnea of prematurity [45] and bronchopulmonary dysplasia (BPD) [46] in the preterm neonates. Recently, caffeine has become a part of the therapeutic protocol in the preterm infants with respiratory distress syndrome (RDS) [47]. Similarly to previously mentioned xanthines, caffeine also possesses anti-inflammatory properties [48].

### 4.2. Nonselective PDE Inhibitors in Experimental Models of ALI

Effects of nonselective PDE inhibitors have been evaluated in various animal models of ALI. Experimental studies of post-treatment with PDE inhibitors are listed in Table 2. Pretreatment with theophylline inhibited neutrophil recruitment and TNFα release in the bronchoalveolar lavage fluid (BALF) of rats with lipopolysaccharide (LPS)-induced ALI [49].

In isolated rabbit lung, pretreatment with aminophylline reduced pulmonary hypertension and lung edema production in both HCl-induced [50] and phorbol myristate acetate (PMA)-induced ALI [51]. In septic guinea pigs, aminophylline pretreatment decreased an albumin leak; however, it decreased also a mean arterial pressure compared to non-treated septic group [52]. Treatment with aminophylline was effective in the rabbit lung exposed to phosgene where it lowered pulmonary artery pressure, tracheal pressure, and lung edema, decreased levels of thiobarbituric acid-reactive substances (TBARS), a marker of lipid peroxidation, and of leukotriene (LT)C4/D4/E4, that stimulates pulmonary capillary permeability, and prevented a phosgene-induced decrease in the lung cAMP [53]. Contrary, in spontaneously breathing rats with endotoxemia, aminophylline at a dose of 1 mg/kg failed to prevent the respiratory and hemodynamic manifestations of sepsis [54]. In rabbits with ALI induced by intratracheal instillation of neonatal meconium, aminophylline administered at two doses at 0.5 h and 2.5 h after meconium instillation enhanced gas exchange, and decreased right-to-left pulmonary shunts, central venous pressure, and ventilatory pressures. Moreover, aminophylline reduced lung edema formation, airway hyperreactivity to histamine, neutrophil count in BALF, and diminished oxidative modifications of proteins and lipids in the lung tissue [55]. Aminophylline at a dose of 2.0 mg/kg demonstrated a superior enhancement in gas exchange, pulmonary shunts, ventilatory pressures, edema formation, and lung neutrophils count in comparison to a lower-dose (1.0 mg/kg) aminophylline, while both dosages decreased lung peroxidation [56]. An improvement in oxygenation and a trend to decrease several markers of inflammation were recently demonstrated also in meconium-instilled newborn piglets [57]. 

Positive results were also observed when using pentoxifylline. Pretreatment with pentoxifylline effectively prevented increases in lung water, albumin lung-to-plasma ratio, markers of inflammation, and oxidative stress in various models of ALI or sepsis [52,58,59,60,61]. Similarly, early treatment with pentoxifylline or with dibutyryl (db)-cAMP, a cell-permeable cAMP analog that is intracellularly deacetylated into cAMP, attenuated several signs of ALI in septic guinea pigs [62]. In pigs with Gram-negative sepsis, both pretreatment and post-treatment with pentoxifylline diminished a lung injury and improved an arterial PaO_2_, while a cardiac index improved when pentoxifylline was given prior to the onset of sepsis or 1 h after the onset of bacteria infusion, but not in a developed septic shock. Plasma TNF activity, blood polymorphonuclears (PMNs), and CD18 (integrin beta chain-2) expression were unaffected by pentoxifylline [63]. In a peritoneal sepsis in rats, a low-dose pentoxifylline suppressed production of IL-6 mRNA in liver and LPS-binding protein mRNA in liver and intestine, which was associated with an improved survival [64]. Pentoxifylline-suppressed production of pro-inflammatory cytokines in the intestine was observed in rats with LPS-induced sepsis, too [65]. Similarly, pentoxifylline decreased local and systemic inflammation, reduced mortality [66,67] and increased levels of antioxidant enzymes in the lung and kidney [68] in rat models of LPS-induced endotoxemia or cecal ligation and puncture (CLP)-induced sepsis, respectively. Favorable effects of pentoxifylline were also demonstrated in models of direct ALI where pentoxifylline prevented an increase in TNFα, protein concentration, and counts of alveolar macrophages in BALF in meconium-injured piglets [69], and reduced histological signs of lung injury and inflammation, diminished oxidative stress, and upregulated markers of wound repair in nitrogen mustard-induced rat model of ALI [70].

Some improvement was also published for pretreatment with lisofylline that decreased a lung leak and lipid alterations, without decreasing neutrophil accumulation in rats with intratracheal IL-1-induced ALI [71]. In minipigs with sepsis, lisofylline pretreatment and treatment given 1 h after injection of bacteria attenuated sepsis-induced pulmonary hypertension, neutropenia, and hypoxemia, and reduced lung edema markers, TBARS concentration, and myeloperoxidase (MPO) activity, but these effects were not seen in the treatment given 2 h after the bacteria [72]. Similarly, a sepsis-induced neutrophil attachment to fibronectin and phagocytic activity were reduced only in the lisofylline pretreatment group, not in the post-treatment groups [73].

Effects of caffeine have been intensively tested in various models of neonatal lung injury and inflammation due to hyperoxia which serve as experimental models of BPD in the preterm neonates. In these studies, caffeine protected from a pulmonary inflammation while it attenuated chemokine and cytokine upregulation and influx of leukocytes [74], elevated cAMP levels, and enhanced alveolar structure and angiogenesis in the lung [75], attenuated the hyperoxia-induced alveolar injury and endoplasmic reticulum stress, and suppressed the activation of cyclooxygenase (COX)-2 and markers of apoptosis [76]. Due to complex anti-oxidant, anti-inflammatory, and anti-apoptotic properties, caffeine may also exert neuroprotective effects on the developing brain in premature neonates [77]. However, caffeine appeared to be beneficial in models of ALI, as well. For instance, chronic caffeine administration for 2 weeks before oleic-acid induced ALI as well as high-dose acute administration 30 min before ALI attenuated the lung edema, hemorrhage, neutrophil recruitment, and expressions of TNFα and IL-1β in both wild type and adenosine A_2A_ receptor knockout mice. This was accompanied by increased cAMP levels and upregulation of A_2B_ receptor mRNA in the lung [78]. Caffeine also inhibited Smad signaling and transforming growth factor (TGF)-β1 regulated genes involved in the airway remodeling, suggesting its potential in diseases with fibrotic changes [79].

Derivatives of caffeine might be also useful. Pretreatment with ***1,7-dimethylxanthine*** (or paraxanthine) inhibited a PARP-1 activity that plays an important role in LPS-induced lung inflammation, lowered lung MPO levels, transcription of IL-6, TNFα, macrophage inflammatory protein (MIP)1α and MIP2 genes and PAR-polymer formation, and reduced markers of systemic inflammation (serum amyloid P component and IL-6) in LPS-induced ALI [48].

### 4.3. Nonselective PDE Inhibitors in Patients with ARDS

Several nonselective PDE inhibitors have been used in patients with ARDS or sepsis, (available studies are listed in Table 3). For instance, early administration of aminophylline and introduction of mechanical ventilation with PEEP in critically-ill patients with ARDS exerted an improvement in oxygenation and Acute Physiology and Chronic Health Evaluation (APACHE) score, and decreased serum epidermal growth factor (EGF), a marker of inflammation [80].

Other nonselective PDE inhibitor pentoxifylline enhanced hemodynamic parameters, i.e., increased heart rate and cardiac index, and decreased systemic vascular resistance index and peripheral vascular resistance in septic patients, what was accompanied by an increase in the oxygen transport and oxygen uptake with unchanged oxygen extraction ratio [81]. Analysis of data from six small studies performed by Cochrane Database System showed that pentoxifylline decreased mortality and length of hospital stay also in patients with neonatal sepsis without any adverse effects [82]. In addition, pentoxifylline may exert broad perspectives in other neonatal disorders, such as meconium aspiration syndrome and hypoxic ischemic encephalopathy in the term neonates, or sepsis [83,84], necrotizing enterocolitis [82,83], and chronic lung disease [85] in the preterm neonates because of its potent anti-inflammatory properties, unique hemorrheologic effects, and very rare adverse effects [86]. In the in vitro measurements, pentoxifylline suppressed LPS-induced and TLR4-mediated cytokine production in a concentration-dependent manner, decreased signaling, and suppressed phagocytosis [87,88]. Despite its favorable effects, pentoxifylline was rarely used in ARDS or severe acute respiratory syndrome (SARS) in the last decades [89,90]. However, within the last year the situation has dramatically changed and an increasing number of articles has been published on the potential benefits of pentoxifylline for treatment of COVID-19. Rationale for the use of PDE inhibitors including pentoxifylline in COVID-19 is discussed in detail in Chapter 7.

Other nonselective PDE inhibitor used in the clinical trials is lisofylline. In a small group of septic patients, lisofylline reduced an ARDS-induced increase in the ratio of serum free fatty acids (i.e., the ratio of C18 unsaturated fatty acids linoleate and oleate to fully saturated palmitate) [91]. However, although the prospective, randomized, placebo-controlled, multicenter study showed a decrease in circulating free fatty acids, it found no additional benefit in the ARDS patients and has been earlier discontinued [92]. From this reason, lisofylline is actually not recommended for the treatment of ARDS [26,93].

## 5. Selective Inhibitors of PDEs

### 5.1. Therapeutic Properties of Selective PDE Inhibitors

As mentioned before, several PDEs are ubiquitously distributed in the cells, which participate in the acute lung damage, i.e., in the lung epithelial and endothelial cells, airway smooth muscle cells, platelets, and immune cells. Considering the distribution of PDEs, PDE3, PDE4, and PDE5 inhibitors might be of a particular benefit in the treatment of ALI/ARDS; however, some improvements might be also demonstrated for other PDE inhibitors. The characteristics of those PDE inhibitors, which might be beneficial in the lung injury, are provided below.

#### 5.1.1. PDE1 Inhibitors

There are three genes known for PDE1. PDE1A gene is highly expressed in the brain but also in the lung tissue and vascular smooth muscle where it regulates the contraction. PDE1B is present in the brain, heart, and skeletal muscle, but also in T-lymphocytes where it participates in IL-13 regulation implicated in allergic diseases and in regulation of apoptosis. PDE1C is highly expressed in proliferating smooth muscle cells [34,38]. PDE1 inhibitors are multi-action agents that target PDE1 modulate vasoconstriction, vascular and cardiac remodeling, and neurotransmission, targeting voltage-gated Na+ channels participate in the regulation of cell toxicity and death, and targeting IKK influence a cellular inflammatory response [94]. Anti-inflammatory effects and effects on microglia signaling [95] support the use of PDE1 inhibitors in the treatment of neurodegenerative diseases and cognitive dysfunction [96]. Mitigation of the vascular remodeling and vasoconstriction [97,98] indicate that PDE1 inhibitors may modulate pulmonary vasoconstriction and smooth muscle proliferation associated with ARDS, as well. In addition, PDE1 inhibitors may exert lung anti-fibrotic effects [99], increase a ciliary beat frequency [39,100], suppress a cough [101], and inhibit a production of pro-inflammatory cytokines and oxidants [102,103], and thereby their use might be of benefit in patients with ARDS.

#### 5.1.2. PDE2 Inhibitors

PDE2 enzymes have been also found in various tissues and cells, including pulmonary arterial smooth muscle cells, endothelial cells, platelets, and macrophages [104]. In the cardiovascular system, PDE2 serves as a regulator for the crosstalk between cAMP/cGMP pathways, which may couple adverse augmented cAMP signaling with cardioprotective cGMP signaling, and thereby PDE2 may serve as a therapeutic target in several cardiovascular diseases [105]. PDE2 inhibitors may be included in the treatment of cognitive disorders, especially anxiety and depression, and other neurodegenerative diseases [106]. Besides other actions, PDE2 likely participates in the regulation of endothelial permeability, endothelial cell proliferation, and platelet functions [34,107].

#### 5.1.3. PDE3 Inhibitors

PDE3 has two main isoforms: PDE3A is mainly present in the heart, platelets, and vascular smooth muscle, while PDE3B was found in the adipocytes, hepatocytes, cardiomyocytes, and spermatocytes [34]. PDE3 inhibitors exert positive inotropic (via PDE3A), vasodilating and weak chronotropic effects, but targeting PDE3B could be beneficial in ischemia/reperfusion injury [108]. PDE3 is present also in the platelets, therefore, PDE3 inhibitors suppress a platelet activation and aggregation [107]. In addition, PDE3 inhibitors are potent modulators of inflammation as it was demonstrated in suppressed inflammatory changes associated with myocardial ischemia–reperfusion [109], or in animal models of asthma, where PDE3 inhibitors reduced the allergic airway inflammation [110], allergen-induced airway hyperreactivity, and cough [111,112]. PDE3 inhibitors also mitigated a serotonin-induced pulmonary hypertension [113,114].

#### 5.1.4. PDE4 Inhibitors

Four genes of the PDE4 family (PDE4A-D) are distributed in various tissues and cells while all of them are present in the lung, and except for PDE4C, they are also present in the inflammatory cells. PDE4 inhibitors exert wide anti-inflammatory effects because of reduced activation and recruitment of inflammatory cells (neutrophils, eosinophils, lymphocytes, monocytes, macrophages, etc.) and related cytokines, as well as reduced release of cytokines and other biologically active substances from the lung structural cells (alveolar and bronchial epithelial cells, microvascular endothelial cells, airway smooth muscle cells, fibroblasts, etc.) [104]. PDE4 inhibitors are also effective in suppressing epithelial-to-mesenchymal transition leading to small airway remodeling [115], in reducing fibroblast pro-fibrotic activation and collagen accumulation, and in decreasing concentrations of chemokines related to fibrosis [116]. In addition, PDE4 inhibitors diminish the airway inflammation airway hyperreactivity, and cough [112,117], and improve the ciliary motility [39,118].

#### 5.1.5. PDE5 Inhibitors

Three variants of PDE5A gene were detected in the heart, kidney, skeletal muscle, pancreas, lung, etc. In the lung, PDE5A is highly expressed in the airway and vascular smooth muscle. Therefore, PDE5 inhibitors act as potent pulmonary vasodilators including hypoxic conditions, and inhibitors of vascular hypertrophy and remodeling [119,120]. In addition, PDE5 inhibitors induce airway relaxation and alleviate inflammation and oxidative stress [117,121,122].

#### 5.1.6. PDE7 Inhibitors

PDE7A1 gene is distributed in T-lymphocytes, eosinophils, epithelial cells, lung fibroblasts, airway, and vascular smooth muscle cells, spleen, lymphatic nodes, etc. PDE7A2 is present in the heart, kidney, and skeletal muscle, PDE7A3 in the immune system, skeletal muscle, heart, and testis, and PDE7B gene in the brain, heart, pancreas, skeletal muscle, and liver [34,104]. Because of wide distribution of PDE7A in the lung and in immune cells, PDE7 inhibitors may be of benefit in inflammatory diseases, particularly in those mediated by T-lymphocytes [123]. Another important property of PDE7 inhibitors is their ability to enhance the effects of PDE4 inhibitors. Additive or even synergistic effects of PDE4 plus PDE7 inhibitors or dual PDE4/PDE7 inhibitors may enable to use lower doses, and thereby reduce the side effects in the same or higher anti-inflammatory and bronchodilator effects [124,125,126].

#### 5.1.7. PDE8 Inhibitors

The PDE8A gene is found in testis, liver, and heart; the PDE8B gene is predominantly present in the thyroid gland and brain. Expression of both PDE8 isoforms may be detected also in the lung, but expressions are low. Nevertheless, expression of PDE8 in the airway smooth muscle cells and T-lymphocytes indicate that inhibition of PDE8 represents an additional target in T-cell-mediated inflammation and bronchoconstriction [127,128].

#### 5.1.8. PDE9 Inhibitors

The PDE9A gene is expressed mainly in the spleen, brain, and small intestine. However, finding of PDE9 in the tracheal smooth muscle and in the immune tissues [34,129] indicate a future possibility of their use in the respiratory disorders.

### 5.2. Selective PDE Inhibitors in Experimental Models of ALI

#### 5.2.1. PDE1 Inhibitors

PDE1 inhibitor vinpocetine inhibited TNFα-induced nuclear factor (NF)-κB activation and subsequent induction of pro-inflammatory mediators in multiple cell types in vitro, including vascular smooth muscle cells, endothelial cells, macrophages, and epithelial cells, and inhibited monocyte adhesion and chemotaxis. Moreover, in vivo vinpocetine pretreatment 2 h before an insult suppressed TNFα- or LPS-induced upregulation of pro-inflammatory mediators, such as TNFα, IL-1β, and MIP-2, and decreased interstitial infiltration of PMNs in these murine models of lung inflammation [130]. In LPS-injured mice, pretreatment with vinpocetine alleviated inflammation-associated hyperalgesia, and MPO activity (as a neutrophil marker), and inhibited neutrophil and mononuclear cell recruitment, production of oxidative stress markers and cytokines (TNFα, IL-1β, and IL-33) [131].

#### 5.2.2. PDE2 Inhibitors

Selective inhibition of PDE2 suppressed a pneumococcal exotoxin pneumolysin-induced hyperpermeability in isolated perfused murine lungs and in human umbilical vein endothelial cell monolayers. Similarly, in a murine model of pneumococcal pneumonia, lung PDE2-mRNA and -protein expressions were increased while a pretreatment with PDE2 inhibitor hydroxy-PDP 1 h before *Streptococcus pneumoniae* infection reduced the vascular permeability [132]. Another PDE2 inhibitor, BAY 60-7550, prevented an onset of both hypoxia- and bleomycin-induced pulmonary hypertension, reduced disease severity [133], and improved mitochondrial respiration and myocardial efficiency in a murine model of intra-abdominal sepsis [134].

#### 5.2.3. PDE3 Inhibitors

As mentioned above, PDE3 inhibitors enhance myocardial contraction and vasodilation. In a porcine model of endotoxemia, endotoxin decreased the cardiac index and hepatic oxygen delivery (DO_2_H), while oxygen consumption (VO_2_H) was unchanged. Treatment with PDE3 inhibitor olprinone unchanged cardiac index, but elevated DO_2_H and VO_2_H indicating a restoration of hepatic circulation, and prevented LPS-induced reduction of cytochrome aa3 and increase in arterial lactate values [135] (Table 4). Vasodilating effects of olprinone on peripheral and pulmonary vessels in hypoxic conditions were shown in a canine model of hypoxic pulmonary hypertension where intravenous olprinone increased heart rate and decreased mean aortic pressure, mean pulmonary arterial pressure, pulmonary vascular resistance, systemic vascular resistance, and right ventricular stroke work index, without the change in cardiac index [136].

However, increasing cAMP PDE3 inhibitors may also modulate an inflammation. Pretreatment with olprinone in a rat model of LPS-induced endotoxemia inhibited neutrophil influx into the lung, suppressed pro-inflammatory cytokines TNFα and IL-6, and increased anti-inflammatory cytokine IL-10 [137]. Olprinone given immediately after induction of sepsis in mice prevented a development of sepsis-associated ALI and improved a survival of septic animals [138]. Similarly, in zymosan-induced multiple organ failure in mice, treatment with olprinone decreased the peritoneal exudation and migration of PMNs. Olprinone also reduced the lung, liver, renal, and pancreatic injury, diminished lung and intestine MPO activity, immunohistochemical staining for nitrotyrosine, inducible NO synthase, PAR, TNFα and IL-1β in the tissue sections obtained from zymosan-injected mice, and prevented systemic toxicity, loss in body weight, and mortality [139]. In CLP-induced sepsis in rats, milrinone elevated antioxidant enzymes and reduced pathological score in the lung and kidney [68].

Favorable effects of PDE3 inhibitors were also demonstrated in meconium-induced ALI with a significant pulmonary hypertension. In rabbits, intravenous olprinone decreased PMNs in BALF, oxidation markers in the lung, and lung edema formation, and prevented a decrease in total antioxidant status (TAS) in the lung. In the plasma, olprinone decreased TBARS and increased total antioxidant status (TAS) [140]. Similarly in newborn piglets, intravenous milrinone improved oxygenation and showed a trend to decrease several markers of inflammation [57]. In other form of direct ALI induced by saline lung lavage in rabbits, olprinone reduced a leak of PMNs into the lung, decreased lung edema production, improved oxygenation, and reduced right-to-left pulmonary shunts. In addition, olprinone decreased pro-inflammatory cytokines IL-1β and IL-6 and a marker of lung epithelial injury RAGE, increased anti-inflammatory IL-10, prevented a decrease in TAS, diminished markers of oxidative stress (TBARS, 3-nitrotyrosine) in the lung, and decreased apoptosis of the lung epithelial cells [141].

#### 5.2.4. PDE4 Inhibitors

Among all PDE inhibitors, PDE4 inhibitors seem to be the most promising in the treatment of ALI/ARDS (Table 4). PDE4 inhibitors stabilize both the endothelial cells and pulmonary epithelium, and thereby reduce the sepsis- and inflammation-induced elevation in a microvascular permeability [142], as it has been demonstrated in several animal in vivo studies. Increasing endothelial cAMP the systemic administration of PDE4 inhibitors rolipram and roflumilast attenuated a capillary leakage and stabilized endothelial barrier properties in the mesenteric venules in LPS-induced systemic inflammation in rats [143]. In the inhaled LPS-induced model of acute pulmonary inflammation, treatment with roflumilast and rolipram decreased transendothelial and transepithelial cell migration, whereas roflumilast showed a superior effect on the epithelium [144]. In a rat model of sepsis, rolipram stabilized a microvascular barrier and improved a microcirculatory flow at a low-dose (1 mg/kg/h), while a high-dose (3 mg/kg/h) was associated with adverse effects [145].

PDE4 inhibitors might be also beneficial in preventing progression of the lung fibrosis. They suppress lung fibroblast proliferation and differentiation into myofibroblasts, the pathological key events in the development of lung fibrosis [146,147], while in vitro measurements highlighted the predominant role of PDE4B in controlling human lung fibroblasts [148]. For instance, roflumilast alleviated bleomycin-induced lung fibrotic responses in mice or rats in a preventive, but also in a therapeutic protocol when roflumilast was administered from day 10 in fully-developed inflammation [149]. In mice with a model of bleomycin-induced injury, other PDE4 inhibitor cilomilast attenuated a late stage of experimental fibrosis when it decreased a fibrosis degree, tended to restore a lung collagen, and increased a lung compliance, but showed no effect on the expression of remodeling markers, such as TGF-β1 and collagen type Ia1 [150]. Roflumilast was also shown to prevent several metabolic effects associated to pulmonary fibrosis, such as alterations in the oxidative equilibrium, strong inflammatory response, and activation of the collagen synthesis [151]. A comparison of an anti-fibrotic efficacy of several PDE4 inhibitors to the two therapies approved by Food and Drug Administration (FDA) for idiopathic pulmonary fibrosis (pirfenidone and nintedanib) showed an equivalent reduction in the lung fibrosis with PDE4 inhibitors to pirfenidone and nintedanib, with a decrease in plasma levels of surfactant protein SP-D and of several chemokines implicated in lung fibrosis, and an in vitro inhibition of fibroblast profibrotic gene expression [116]. Anti-fibrotic action of PDE4 inhibitors was also demonstrated in the study by Fehrholz et al. [79] where rolipram inhibited Smad signaling and TGF-β1 regulated genes involved in the airway remodeling in a concentration-dependent manner. In agreement with these studies, PDE4 inhibitors were of benefit also in a hyperoxia-induced model of neonatal BPD. Treatment with rolipram and piclamilast prolonged a median survival, reduced alveolar fibrin deposition, lung inflammation, and vascular leakage, and reduced key genes involved in inflammation, fibrin deposition, and alveolarization [152].

PDE4 inhibitors exert a potent anti-inflammatory activity, as well. As previously mentioned, PDE4B seems to be predominantly related to an inflammation. The study by Ma et al. showed that cilomilast and PDE4B knockout inhibited the LPS-induced NF-κB activation and inflammatory response in multiple cell types, including lung epithelial cells, pulmonary microvascular endothelial cells and vascular smooth muscle cells [153]. In addition, PDE4B deletion attenuated the LPS-induced ROS generation [153]. In vivo, PDE4B deletion reduced LPS-induced vascular permeability and histological signs of lung injury, and elevated the PaO_2_/FiO_2_ ratio [153]. These results were supported by the study by Konrad et al. who found that inhaled LPS enhanced expression of both PDE4B and PDE4D in the lung, whereas PDE4 inhibitors decreased mainly the PDE4B [144].

Pretreatment with PDE4 inhibitors obviously reduced a cell sequestration in a dose-dependent manner [154,155]. Similarly, a pretreatment with other inhaled PDE4 inhibitor GSK256066 effectively inhibited a LPS-induced pulmonary neutrophilia and an increase in exhaled NO in rat and ferret models of acute pulmonary inflammation [156]. Favorable effects of PDE4 inhibitors delivered after the induction of lung injury have been demonstrated in a number of studies, including bleomycin-induced lung fibrosis models. In an early (inflammatory) phase of the lung fibrosis model, cilomilast reduced the count of alveolar inflammatory cells and the counts of macrophages and lymphocytes, but not neutrophils in BALF, decreased lung TNFα mRNA level, and increased IL-6 mRNA level, but did not influence IL-1β [150], and roflumilast diminished lung transcriptions for TNFα, and reduced BALF levels of TNFα, IL-13, lipid hydroperoxides, and inflammatory cell counts [149]. Systemic delivery of PDE4 inhibitors effectively attenuated inflammation in other models of direct ALI, as well. In inhaled LPS-induced ALI, intraperitoneally given roflumilast and rolipram decreased TNFα, IL6, and CXCL2/3, while CXCL1, a potent PMN chemoattractant released by the epithelium, was more suppressed by roflumilast. Predominantly roflumilast decreased LPS-induced stress fibers, essential for a direct migration of PMNs into the alveolar space [144]. In a saline lavage-induced ALI in rabbits, intravenous roflumilast decreased a leak of cells, particularly of neutrophils, into the lung, declined concentrations of cytokines and oxidation products in the lung and plasma, prevented lung cell apoptosis and edema formation, and enhanced gas exchange [157,158]. Intravenous rolipram was also beneficial in a rat model of polymicrobial sepsis where it decreased IL-1α, IL-1β, IL-12, and TNFα levels [145].

Importantly, PDE4 inhibitors may mitigate the ALI- or sepsis-associated injury to the liver, heart or kidneys. Stabilization of endothelial cAMP levels prevents the loss of microvascular endothelial barrier function and dysfunctional microcirculation, which both promote an organ failure in sepsis [159]. PDE inhibitors may also protect from a sepsis-induced heart dysfunction due to blunted mitochondrial cAMP-PKA pathway [134]. In LPS-induced ALI in rats, pretreatment with rolipram inhibited LPS-induced alterations in renal and hepatic function, indicated by increased blood urea nitrogen, alanine aminotransferase (ALT), and aspartate aminotransferase (AST) [160]. Systemic treatment with rolipram in rats with polymicrobial sepsis prevented a sepsis-induced acute kidney injury and lung edema [145]. In a murine model of sepsis, rolipram acutely restored a capillary perfusion, enhanced a renal blood flow, and reduced an increased renal microvascular permeability while delayed treatment with rolipram at 6 h after induction of sepsis restored the renal microcirculation, decreased blood urea nitrogen and serum creatinine, and elevated glomerular filtration rate at 18 h [161]. In a rat model of infant sepsis associated with a development of both acute cardiac dysfunction and acute kidney injury, administration of rolipram improved a 4-days survival, restored cardiac function and renal microcirculation, and decreased a microvascular leakage [162].

As mentioned above, PDE4 in the lung has been extensively studied in the airway smooth muscle. However, PDE4 is also present in the pulmonary artery smooth muscle cells and an exposure to hypoxia increases an expression of several PDE4 isoforms [163]. For this reason, PDE4 inhibitors might be of benefit in situations, such as persistent pulmonary hypertension of the newborn (PPHN) or in abnormally constricted pulmonary vasculature in sepsis, RDS, meconium aspiration syndrome etc., as well as in ARDS-induced pulmonary vasoconstriction and pulmonary hypertension, although the PDE5 and PDE3 inhibitors are of a major importance in influencing of these changes [164].

#### 5.2.5. PDE5 Inhibitors

A potential benefit of PDE5 inhibitors results from their ability to modulate the ARDS-associated alterations in the pulmonary blood flow and lung microcirculation, as well as the procoagulant and thrombotic events in the pulmonary arteries [165] (Table 4).

Potent pulmonary vasodilation effect of PDE5 inhibitors was shown in several studies. For instance, sildenafil reduced acute hypoxic pulmonary vasoconstriction while continuous administration of sildenafil attenuated the hypertrophy of right ventricle and pulmonary vascular remodeling in the isolated perfused murine lung chronically exposed to hypoxia [166]. Oral delivery of other PDE5 inhibitor tadalafil in a hypoxia-induced piglet model of neonatal PPHN decreased a pulmonary arterial pressure on average by 54%, increased a cardiac output by 88%, and increased a PaO_2_ as a result of reduction in the alveolar-arterial oxygen gradient expressing reduced lung shunt fraction [167].

Positive effects of PDE5 inhibitors have been also demonstrated in animal models of direct or indirect lung injury. In the direct lung injury, for example, sildenafil treatment decreased a leak of cells, particularly of neutrophils, into the lung, suppressed a release of pro-inflammatory mediators (TNFα, IL-8 and IL-6) and markers of oxidative damage, reduced lung edema formation and apoptosis of epithelial cells, and enhanced respiratory parameters in rabbits with saline lavage-induced model of ALI [168]. In a model of ALI-associated pulmonary hypertension induced in piglets by endotracheal instillation of meconium, sildenafil reversed an increase in the pulmonary vascular resistance induced by meconium within 1 h of sildenafil infusion, whereas no effect on the systemic hemodynamics was observed. Sildenafil also improved cardiac output, which was not accompanied by a deterioration in oxygenation [169]. Increasing doses of sildenafil in piglets with meconium-induced pulmonary hypertension reduced pulmonary artery pressure and pulmonary vascular resistance, however, caused a dose-related increase in oxygenation index [170].

Nevertheless, PDE5 inhibitors have been effective in the indirect lung injury, as well. In a model of CLP-induced sepsis in rats, administration of sildenafil 8 h after the insult elevated a renal blood flow and decreased the plasma levels of creatinine, lactate, and creatine kinase, and lung myeloperoxidase [171]. In acute pancreatitis-associated ALI in rats sildenafil reduced an extent of the lung injury and inflammation, elevated an expression of proliferation-related markers, and suppressed an expression of apoptosis-related markers [172]. Sildenafil was also beneficial in a severe scald burn-induced ALI model in rats where it decreased markers of oxidative stress and lung inflammation scores, and increased an anti-oxidative capacity [173]. In addition, sildenafil treatment by decreasing inflammation and oxidative stress prevented an injury in remote organs (liver and kidneys) [174]. Protective effects of sildenafil on the lung and extrapulmonary organs were exerted in a polymicrobial sepsis in rats where sildenafil increased total glutathione (GSH), and decreased an activation of MPO, and decreased malondialdehyde (MDA) as a marker of lipid peroxidase, and superoxide dismutase (SOD) in the lung and kidney of septic rats. In addition, sildenafil decreased the serum TNFα and a higher dose of sildenafil (20 mg/kg) enhanced histopathological inflammation scores [175].

Promising results may be also obtained from the use of dual PDE inhibitors. For instance, dual PDE4/PDE5 inhibitor LASSBio596 prevented the changes in lung mechanics, and suppressed the neutrophilic recruitment, TNFα release, bronchoconstriction, alveolar collapse, and increase of collagen fiber content induced by intratracheal LPS [176].

#### 5.2.6. PDE7 Inhibitors

Despite potential perspectives of PDE7 inhibitors in the treatment of ALI, only one study has been recently published on this topic. PDE7 inhibitor GRMS-55 delivered simultaneously with LPS decreased a plasma concentration of TNFα in an acute endotoxemia in rats induced by intravenous LPS [177].

### 5.3. Selective PDE Inhibitors in Patients with ARDS

Despite encouraging results from a preclinical testing, the selective PDE inhibitors are used sporadically in patients with ARDS or sepsis (Table 3), except for the ongoing studies on COVID-19 (see Chapter 7).

Up to now, there have been no studies available on the use of PDE1 or PDE2 inhibitors in the clinical studies carried out on patients with severe respiratory distress related to primary or secondary lung injury.

A better situation is in case of PDE3 inhibitors, as these drugs may exert some benefits in the treatment of PPHN [164,178]. However, there are only two clinical studies demonstrating the use of PDE3 inhibitors in ARDS or sepsis. In the study by Barton et al., intravenous milrinone given to pediatric patients with non-hyperdynamic septic shock enhanced hemodynamic parameters, i.e., increased cardiac index, stroke volume index, and DO_2_, and decreased systemic vascular resistance index, pulmonary vascular resistance index, and mean pulmonary arterial pressure, without any serious adverse effects [179]. In the study by Wang et al., the milrinone-only therapy but especially the combination of milrinone plus β-blocker esmolol treatment improved the cardiac function and 28-day survival rates, reduced heart rate, and inhibited the inflammatory response [180].

Similarly to PDE3 inhibitors, PDE5 inhibitors, particularly dipyridamole, sildenafil, and tadalafil, have been used in the treatment of PPHN [164,181,182] and pulmonary hypertension of different origin including hypoxemic pulmonary hypertension [183,184,185,186]. In patients with ARDS-associated pulmonary hypertension, sildenafil decreased mean pulmonary arterial pressure and pulmonary artery occlusion pressure, but decreased systemic mean arterial pressure and increased the shunt fraction [187].

## 6. Limitations of the Use of PDE Inhibitors

There are several well-known adverse effects of PDE inhibitors, which can reduce their broader use. A majority of adverse effects of theophylline or other xanthines is probably related to an adenosine receptor antagonism and it might be avoided by monitoring the plasma levels [37]. The therapeutic doses of theophylline range from 3 to 6 mg/kg supplying serum levels of 10 to 20 μg/ml, which are necessary for bronchodilation effect. Toxic effects of theophylline on gastrointestinal system (e.g., nausea, vomiting, diarrhea), and central nervous system (e.g., insomnia, irritability, headache) were described at serum concentrations over 20 μg/ml. At concentrations greater than 30 μg/ml serious neurological or cardiovascular symptoms might occur [36]. Both theophylline and aminophylline increase the cardiac muscle contractility and efficiency (positive inotropic effect) and thereby increase the blood pressure, and increase the heart rate (positive chronotropic effect) what might be related to antagonism of adenosine receptors, PDE3 inhibition, and increased plasma levels of epinephrine and norepinephrine [188,189,190].

Among the selective PDE inhibitors, adverse effects have been intensively discussed for PDE4 inhibitors. In contrast to theophylline, administration of PDE4 inhibitors does not require a plasma monitoring and exerts less interactions with other drugs. However, use of PDE4 inhibitors is associated with a higher occurrence of gastrointestinal symptoms, such as nausea, vomiting, diarrhea, and abdominal pain, which limits their wider use [191]. To avoid or minimize the adverse effects of PDE4 inhibitors, there exist several strategies: to prefer inhalational forms (what might be associated with a decrease in effectiveness), to dissociate beneficial and detrimental effects, e.g., by the use of derivatives with lower penetration to central nervous system, by selective targeting the PDE4 isoenzymes (selectively targeting PDE4B, not PDE4D, which is responsible for gastrointestinal adverse effects), or to search for allosteric inhibitors [125,192].

Among the most important adverse effects of selective PDE5 inhibitors belong headache, flushing, nasal congestion, nasopharyngitis, dyspepsia, as well as rarer but more severe, e.g., vision changes, myalgia, and seizures [193].

## 7. PDE Inhibitors in SARS-CoV2-Induced ARDS

Recent data show that both nonselective and selective PDE inhibitors might be useful in ARDS caused by a severe infection with severe acute respiratory syndrome coronavirus 2 (SARS-CoV2) leading to COVID-19. Current knowledge on the pathophysiology of COVID-19 and participation of the cyclic nucleotide pathways in inflammation, fibrosis, vascular resistance, thrombosis, and stroke have been recently published by Giorgi et al. in their excellent review [194]. In this chapter, we present a short overview of those COVID-associated changes related to PDEs and provide the data about ongoing clinical studies where the PDE inhibitors have been administered to patients with COVID-19-induced ARDS.

Several changes associated with COVID-19 are closely related to renin–angiotensin–aldosterone (RAS) system. Angiotensin-converting enzyme (ACE) splits angiotensin I to angiotensin II that is known as a potent vasoconstrictor, also responsible for hypoxia-induced pulmonary vasoconstriction and pulmonary edema, a mitogen for smooth muscle cells and fibroblasts, and a promotor of oxidative stress, activation of complement, and of release of pro-inflammatory cytokines, including IL-6, TNFα, etc. Opposite, ACE2 catalyzes the change of angiotensin II to angiotensin 1–7 peptide that acts as a vasodilator, anti-inflammatory, anti-proliferative, anti-fibrotic, and antioxidant agent, and thereby protects from the lung injury [195,196].

Entry of the SARS-CoV2 virus is mediated by a surface spike protein, which binds to the ACE2 receptors on the host cells. ACE2 is highly expressed in the lung alveolar and bronchial membranes, in pneumocytes type II, and possibly on vascular endothelial cells as well as in oral and nasal mucosa, gastrointestinal tract, heart, liver, kidney, skin, brain, etc., which can explain common respiratory and extra-pulmonary symptoms in COVID-19 [197,198]. However, binding of SARS-CoV2 with ACE2 receptor downregulates the ACE2 expression, which increases the levels of angiotensin II exerting the above mentioned deleterious effects to the lung [199]. In addition, binding of SARS-CoV virus on the ACE2 receptors triggers an activation of innate immune reaction to combat an infection, with an activation of various immune cells and a production of enormous concentrations of cytokines, so-called cytokine storm [194]. In the pathophysiology of the SARS-CoV2-induced ARDS, IL-6 is of a particular importance; it promotes a clearance of the virus by neutrophils, triggers an accumulation of fluid and immune cells including neutrophils in the lung, causes a serious endothelial dysfunction, and induces an intestinal, olfactory, ocular inflammation causing diarrhea, anosmia, and conjunctivitis, the extra-pulmonary signs of COVID-19 [199,200]. SARS-CoV2 itself induces an endothelial dysfunction, which results in a platelet activation and aggregation, and finally leads to a pulmonary intravascular coagulopathy and a deep vein thrombosis. The clots cause a compensatory increase in plasminogen (fibrinolysin) levels; however, in advanced course of the disease, it fails to break down the fibrin deposits [201,202]. The structural and functional changes of endothelium and activated coagulation further stimulate a leukocyte trafficking. In the SARS-CoV infection, the inflammation is mediated mainly by neutrophils representing the first line of defense of innate immune system [199]. The activated neutrophils exert phagocytosis, degranulation, and release of proteases (elastase, cathepsin G etc.), pro-inflammatory cytokines (IL-6, TNFα etc.), generation of a respiratory burst with a production of ROS, and a formation of neutrophil extracellular traps (NET). Dysregulated immune responses with excessive mobilization and activation of neutrophils, and, opposite, with decreased counts and reduced activity of lymphocytes responsible for a cell-mediated immunity, are associated with a negative prognosis of patients [203]. Simultaneously, endothelin-1 as a product of injured endothelium exaggerates the inflammation by decreasing cAMP [204] and stimulates a pulmonary fibrosis by increasing the release of TGF-β1 [205]. The multiple effects of dysregulated inflammation, endothelial dysfunction, and hypercoagulopathy may finally lead to the multiorgan damage and failure, with a higher mortality risk in elderly people and in patients with chronic diseases [199]. Extent of the injury to the lung and other organs (heart, liver, muscles, kidneys, etc.) correlates well with elevated serum levels of lactate dehydrogenase (LDH) and increased neutrophil/lymphocyte ratio [206].

Recent publications demonstrate that the use of PDE inhibitors might be a valuable adjunctive treatment approach in the SARS-CoV-induced ARDS [194,203], beside standard interventions, such as lung-protective ventilation, prone positioning, neuromuscular blockade for a patient-ventilator synchrony, maintenance of a negative fluid balance, administration of antibiotics preventing secondary bacterial and fungal infections, and administration of antiviral drugs [207].

For instance, nonselective PDE inhibitor pentoxifylline suppresses the synthesis of cytokines and other molecules involved in inflammation, lowers the neutrophil/lymphocyte ratio restoring Treg/Th17 lymphocyte populations, and decreases the levels of LDH and ferritin with a subsequent enhancement of PaO_2_ and oxygen saturation [31,89,206,208,209]. Pentoxifylline affects the RAS system reducing the expression of angiotensin II receptor type 1 (AT1) that mediates the biological effects of angiotensin II contributing to a severe acute lung injury. Pentoxifylline also participates in a restoration of GSH levels and a mitochondrial viability [208]. In addition, pentoxifylline may preserve a microvascular circulation decreasing a viscosity of blood and increasing a deformability of erythrocytes, improving an endothelial function, and decreasing a platelet aggregation [210]. Pentoxifylline might also reduce a tissue fibrosis *via* reducing an expression of platelet activating factor (PAI)-1 and fibronectin [211] or by blocking TGF-β1 and reducing a deposition of type I collagen [212]. There are several other advantages of the use of pentoxifylline in COVID-19: over 50 years of safety data from its use in other disorders, good tolerability and safety, an available oral form with a good bioavailability, but can be also administered intravenously or inhalationally in the selected patients [213]. In an external pilot study carried out on 38 patients with moderate-to-severe COVID-19, 26 patients were given pentoxifylline orally at a dose of 400 mg every 8 h plus standard therapy. Compared to the control group with standard therapy-only, pentoxifylline treatment increased a lymphocyte count, decreased a serum LDH, and showed a trend toward reduced days of hospitalization, mortality, and requirements for an intubation [206]. Effects of pentoxifylline (400 mg sustained release (SR) three-times a day) have been currently evaluated in the ongoing clinical trial for COVID-19 (NCT04433988).

Because of its ability to inhibit a hemocoagulation, to cause a vasodilation, and to suppress an inflammation including the inhibition of NET formation via increasing cAMP and blocking an adenosine reuptake [203,214], use of PDE3 inhibitor dipyridamole might also be of benefit in COVID-19 patients. In a clinical trial involving 31 patients with COVID-19, 14 patients were treated by dipyridamole (50 mg orally, three-times daily for 14 days). Dipyridamole treatment decreased a level of D-dimers (degradation products of fibrin), enhanced a lymphocyte and platelet recovery in the circulation, and improved a clinical outcome in comparison with the control patients [215]. Effects of dipyridamole in combination with acetylsalicylic acid or of dipyridamole-only have been evaluated in patients with COVID-19 in three ongoing clinical trials: (1) NCT04410328 (ATTAC-19 trial), Aggrenox (dipyridamole extended release (ER) 200 mg/ aspirin 25 mg), given orally/internally twice daily; (2) NCT04424901 (TOLD trial), dipyridamole 100 mg, given three-times a day orally for 7 days; and (3) NCT04391179 (DICER trial), dipyridamole 100 mg, given four-times a day orally for 14 days. The recent article by Motta et al. suggested other PDE3 inhibitor cilostazol as a potentially beneficial drug for the treatment of COVID-19 [216].

Favorable effects in COVID-19 may be also expected for the PDE4 inhibitors which modulate processes in various immune cells including neutrophils and inhibit an activation of NF-κB pathway mediating the production of pro-inflammatory cytokines and ROS [217]. PDE4 inhibitors reduce the leukocyte-platelet interactions and a prothrombotic action of leukocytes, decrease an endothelial permeability, and exert the anti-thrombotic effects [142,218]. In addition, PDE4 inhibitors may reduce fibrosis in the lung by several mechanisms [198]. With regard to worse outcome and higher mortality of COVID-19 in older patients with diabetes mellitus or cardiovascular disorders, PDE4 inhibitors possess an advantage as they are able to increase a PDE4 activity in elderly, to reduce a body weight, to improve an insulin sensitivity [219], and to prevent adverse cardiovascular events of nonselective PDE inhibitors (xanthines) [220].

In light of the above, effects of PDE4 inhibitor apremilast in COVID-19 patients have been evaluated in the ongoing clinical trial named I-SPY_COVID (NCT04488081) where apremilast (30 mg orally, given twice a day for 14 days) has been combined with remdesivir (200 mg loading dose on day 1, followed by 100 mg given intravenously once daily for 4 or 9 days). An eventual additional improvement of adding apremilast to remdesivir on a mortality and a need for mechanical ventilation will be compared to remdesivir-only therapy.

Positive effects of both PDE3 and PDE4 inhibitors in COVID-19 might be demonstrated by the ongoing clinical trial no. NCT04527471, where dual PDE3/PDE4 inhibitor ensifentrine (RPL554) has been administered inhalationally via pressurized metered dose inhaler (pMDI) twice daily. However, recent studies show that its inhibitory effect on PDE4 is only limited [221,222].

Finally, PDE5 inhibitors as modulators of the NO-cGMP-PDE5 axis expressed predominantly in the lung may show a wide spectrum of cardioprotective, anti-aggregation, anti-inflammatory, antioxidant, and beneficial hemodynamic actions. To evaluate the potentially favorable effects in patients with COVID-19, PDE5 inhibitor sildenafil has been administered in two ongoing clinical trials: (1) NCT04304313 (sildenafil citrate 100 mg/day, given orally for 14 days); and (2) NCT04489446 (sildenafil 25 mg every 8 h, given orally for 7 days). The results have not yet been revealed at time of this review.

## 8. Concluding Remarks

Several nonselective and selective PDE inhibitors have successfully taken a part in the treatment of various respiratory, neurological, or cardiovascular diseases. Considering a broad experience with the use of PDE inhibitors in other diseases including their side effects or interactions with other drugs, it is surprising that there are only several clinical studies on acute lung damage where the PDE inhibitors have been used. PDE inhibitors may exert wide advantages in ARDS, particularly due to their ability to suppress an inflammation, to alleviate the edema formation, oxidative stress, and damage to lung epithelium and endothelium, to reduce activation of platelets, and to prevent hypercoagulation state.

The results of experimental and clinical studies indicate that both nonselective and selective PDE inhibitors possess a great potential to improve the course of direct or indirect lung injury in ARDS or sepsis, respectively. Nonselective PDE inhibitors have an advantage of simultaneous modulation of several PDEs at once and, thereby, provide a wider therapeutic impact. On the other hand, this multiple action may be associated with a higher occurrence of harmful reactions. Although the selective PDE inhibitors might theoretically cause less unwanted effects since their actions are focused on one PDE, they cannot be reliably excluded. The choice of the appropriate PDE inhibitor should be conditioned by the general status of the ARDS patient and by the severity and subtype of ARDS. For instance, in the subtype of ARDS with prominent inflammatory component, the PDEs derivative influencing PDEs in the inflammatory cells, e.g., PDE4 inhibitors, could be more effective. In primary damage of the pulmonary capillaries and in hypercoagulation state, more suitable could be those PDEs derivatives modulating PDEs in the platelets and endothelial cells, e.g., PDE3 and PDE5 inhibitors.

Of course, similar to other approaches in PDE inhibitors, it is necessary to consider a value of expected favorable effects versus potential adverse effects of the treatment. Rather big variabilities of the adverse effects of PDE inhibitors results from a heterogenous distribution of individual PDEs in different cells and tissues. Nevertheless, side effects of PDEs are generally less serious than the side effects of other anti-inflammatory and anti-edematous medicines, e.g., corticosteroids.

Despite convincing data and undoubted advantages, use of PDE inhibitors in ARDS is lower than they deserve, likely due to rare or missing clinical studies on the use of nonselective and selective PDEs in the treatment of ARDS or sepsis. However, this unfavorable situation will probably change as there have been several clinical studies carried out on effects of PDE inhibitors in the patients with COVID-19-associated ARDS. Positive results from these ongoing studies could raise interest in PDE inhibitors and their subsequent use in ARDS and sepsis from other than COVID-19 origin, as well.

## Figures and Tables

**Table 1:** Distribution of phosphodiesterases (PDEs), biological effects and examples of inhibitors.

PDEs Family	Substrate	Primary Tissue Distribution of PDEs	Biological Effects of PDEs	Examples of Inhibitors
PDE1	cAMP/cGMP	brain, heart, lung, smooth muscle, adipocyte, pancreas, neurons, macrophages/monocytes, testes/spermatozoa	regulation and proliferation of vascular smooth muscle, survival and activation of immune cells, neuronal functions, spermatogenesis	Vinpocetine Nicardipine nimodipine
PDE2	cAMP/cGMP	adrenal gland, heart, lung, liver, platelets, endothelial cells, adipocyte, brain	regulation of endothelial permeability and platelet functions, regulation of aldosterone secretion, neuronal functions, long-term memory	EHNA BAY-60-7750 oxindole
PDE3	cAMP/cGMP	heart, smooth muscle, lung, liver, platelets, adipocyte, pancreas, immune cells, brain, endothelium, epithelium, oocytes	myocardial contractility, platelet aggregation, vascular and airway smooth muscle contraction, regulation of inflammation and fibrosis, platelet functions, mediation of response to insulin, regulation of cell proliferation	Milrinone cilostazol olprinone ensifentrine (RPL554)
PDE4	cAMP	brain, Sertoli cells, kidney, liver, heart, smooth muscle, lung, adipocyte, endothelial cells, immune cells, pancreas	neuronal functions, regulation of inflammation, microvascular permeability, and fibrosis, vascular and airway smooth muscle contraction and proliferation, myocardial contractility, fertility	rolipram cilomilast roflumilast apremilast
PDE5	cGMP	lung, platelets, smooth muscle, heart, endothelial cells, brain, kidney, gastrointestinal system	vascular and airway smooth muscle contraction and remodeling, platelet functions, participation in inflammation and oxidative stress, neuronal functions	sildenafil dipyridamole zaprinast tadalafilvardenafil
PDE6	cGMP	photoreceptors, pineal gland	signal transmission and photoreaction, regulation of melatonin release	none selective
PDE7	cAMP	skeletal muscle, vascular smooth muscle, lung, cardiomyocytes, kidney, brain, pancreas, T- and B-lymphocytes, spleen	activation of T-lymphocytes and other immune cells, bronchoconstriction	BRL 50481 IC242 ACB16165
PDE8	cAMP	testes, eye, liver, skeletal muscle, heart, kidney, ovary, brain, T-lymphocytes, thyroid gland, pancreas	activation of T-cells, regulation of spermatogenesis and Leydig cells, bronchoconstriction	PF-04957325
PDE9	cGMP	kidney, spleen, liver, lung, brain, intestinal cells, skeletal muscle, heart	neuronal functions, participation in inflammation and bronchoconstriction	BAY-73-6691 PF-04447943
PDE10	cAMP/cGMP	testes, brain, thyroid gland, pancreas	neuronal functions, learning, memory processes	papaverine TP-10 MP-10
PDE11	cAMP/cGMP	skeletal muscle, prostate, kidney, liver, pituitary and salivary glands, testes	spermatogenesis	none selective

**Table 2:** Studies of post-treatment with nonselective PDE inhibitors in animal models of acute respiratory distress syndrome (ARDS) or sepsis.

Author (Year)	Type of Injury	Species of Animals	Treatment/Dose	Duration of Treatment	Outcomes in Treated Groups
Sciuto et al. (1997)	Phosgene-induced ALI (nebulized LPS 100 μg/mL for 60 min.)	Wistar rats	Aminophylline (lung perfusion with 30 mg/kg)	2.5 h	Decreased pulmonary artery pressure, tracheal pressure, and lung weight gain, reduced TBARS and perfusate LTC4/D4/E4, prevented decrease in lung cAMP
Fakioglu et al. (2004)	LPS-induced acute endotoxemia (LPS 4 mg/kg i.v.)	Sprague–Dawley rats	Aminophylline (1 mg/kg i.v. bolus 30 min, then 0.5 mg/kg/h infusion), concomitant injection with LPS)	6 h	No decrease in tissue (lung, kidney, heart) water content
Mokra et al. (2007)	Meconium-induced ALI	Rabbits	Aminophylline (2 mg/kg i.v. bolus 0.5 and 2.5 h after i.t. meconium)	5 h	Improved gas exchange, decreased right-to-left shunts and ventilatory pressures, reduced edema formation, neutrophils in BALF, markers of oxidative stress, airway reactivity to histamine
Mokra et al. (2008)	Meconium-induced ALI	Rabbits	Aminophylline (2 mg/kg or 1 mg/kg i.v. bolus 0.5 and 2.5 h after i.t. meconium)	5 h	With 2 mg/kg A: superior effect on gas exchange, lung edema, lipid peroxidation, neutrophil counts in BALF and tracheal reactivity to histamine; 1 mg/kg A: reduced protein oxidation in the lung and lung tissue reactivity to histamine
Shao et al. (2019)	Meconium-induced ALI	Newborn piglets	Aminophylline (6 mg/kg followed by continuous infusion of 0.5 mg/kg/h)	4 h	Improved oxygenation, trend to decrease markers of inflammation (IL-8 and CRP in BALF, IL-8 and PLA_2_ in lung)
Hoffmann et al. (1991)	*E.coli*-induced sepsis (2 × 10^9^ *Escherichia coli* bacteria i.v.)	Guinea pigs	Pentoxifylline (20 mg/kg i.v. followed by infusion of 20 mg/kg/h) or Dibutyryl (db)-cAMP (2 mg/kg i.v. + 2 mg/kg/h infusion) 30 min. after *E. coli*	7.5 h	Both treatments prevented edema formation; reduced *in vitro* endotoxin-induced PMNs chemiluminescence, no change in leukocyte counts in BALF or peripheral leukocytes
Riddings et al. (1994)	*Pseudomonas aeruginosa*-induced Gram-negative sepsis by i.v. infusion	Yorkshire swine	Pentoxifylline (20 mg/kg followed by 6 mg/kg/h) prior to the onset of sepsis, or at 1 h or 2 h after the onset of bacteria infusion	5 h	Both pre- and post-treatments improved PaO_2_ and lung injury, pre- and early post-treatment improved cardiac index, but late treatment in established septic shock caused fatal systemic hypotension in a half of animals
Nelson et al. (1999)	Peritoneal sepsis	Lewis rats	Pentoxifylline (low dose of 5 mg/kg/day vs. high dose of 20 mg/kg/day)	7 d	Low-dose P: improved survival, down-regulated IL-6 mRNA in liver and LPS-binding protein mRNA in liver and intestine
Ji et al. (2004)	LPS-induced sepsis (LPS 5 mg/kg i.p.)	Rats	Pentoxifylline (doses of 6.25, 12.5, 25, 50, or 100 mg/kg at 1 min after LPS challenge)	1, 4 and 6 h	Pentoxifylline at all dosages reduced activation of NF-κB and production of TNFα and IL-6, and enhanced a release of IL-10
Coimbra et al. (2005)	LPS-induced acute endotoxemia (LPS 5 mg/kg i.v.)	Male Sprague-Dawley rats	Pentoxifylline (25 mg/kg i.v., concomitant injection with LPS)	2, 4 or 24 h	Decreased plasma TNFα, IL-6, nitrite, AST and ALT, reduced liver injury score and neutrophil infiltration, decreased NF-κB in hepatocytes and Kupffer cells, decreased iNOS in Kupffer cells
Coimbra et al. (2006)	LPS-induced acute endotoxemia (LPS 5 mg/kg i.v.)	Male Sprague-Dawley rats	Pentoxifylline (25 mg/kg i.v., concomitant injection with LPS)	4 h	Decreased IL-8 and MMP-2 in BALF, and plasma MMP-9 activity, decreased lung MPO, ICAM-1 expression, and NF-κB activation, attenuated lung injury
Korhonen et al. (2004)	Meconium-induced ALI	Neonatal piglets	Pentoxifylline (20 mg/kg bolus i.v. followed by infusion of 20 mg/kg/h) since 15 min. after meconium	12 h	Enhanced regional ventilation, prevented an increase of alveolar macrophages in BALF and lung, concentrations of TNFα and protein, but had no effect on lung neutrophil accumulation
Sunil et al. (2014)	Nitrogen mustard- induced ALI	Wistar rats	Pentoxifylline (46.7 mg/kg, i.p.) daily for 3d beginning 15 min after nitrogen mustard	3 days	Reduced lung injury, protein content, and cells in BALF, decreased COX-2 and MMP-9, oxidative stress proteins lipocalin and heme-oxygenase-1 in lung, stimulated wound repair
Özer et al. (2020)	CLP-induced model of sepsis	Wistar rats	Pentoxifylline (50/mg/kg/day i.p.) for 72 h after CLP	7 days	Increased antioxidant enzymes and lowered pathological score in the lung and kidney
Hasegawa et al. (1997)	*Pseudomonas aeruginosa*-induced sepsis by i.v. infusion	Hanford minipigs	Lisofylline (25 mg/kg followed by 10 mg/kg/h) 30 min prior to, or 1 h or 2 h after bacteria infusion	6 h	Attenuated sepsis-induced pulmonary hypertension, neutropenia, hypoxemia, increased MPO activity, and lung injury parameters in the Pre- and Post-1 h groups
Oka et al. (1999)	*Pseudomonas aeruginosa*-induced sepsis by i.v. infusion	Hanford minipigs	Lisofylline (25 mg/kg bolus followed by infusion of 10 mg/kg/h) 30 min prior to sepsis, or 1 h or 2 h after bacteria infusion	6 h	Pretreatment reduced sepsis-enhanced phagocytic activity, attenuated neutrophil attachment to fibronectin, and produced neutrophilia; 1-h post-treatment attenuated neutrophil attachment to fibronectin and caused neutropenia to recover
Weichelt et al. (2013)	Hyperoxia-induced ALI	Neonatal rats	Caffeine (10 mg/kg i.p.) at the beginning of hyperoxia	6, 24, or 48 h	Diminished lung leukocyte infiltration, perturbation of alveolar development, and mRNA expressions of pro-inflammatory chemokines and cytokines
Jing et al. (2017)	Hyperoxia-induced ALI	Neonatal rats	Caffeine (20 mg/kg i.p.) from day 2 of life	9 or 20 days	Increased lung cAMP levels and phosphorylated endothelial NO synthase, improved alveolar structure and angiogenesis, reduced mortality
Teng et al. (2017	Hyperoxia-induced ALI	Neonatal rats	Caffeine (20 mg/kg i.p.) from day 2 of life	9 or 20 days	Attenuated alveolar injury and endoplasmic reticulum stress, suppressed activation of COX-2 and markers of apoptosis
Li et al. (2011)	Oleic acid-induced ALI	Wild type and A2A receptor knockout C57BL/6 mice	Caffeine: chronic (0.1 g/l, 0.25 g/l and 5.0 g/l p.o.) for 2 weeks before ALI, or acute (5 mg/kg, 15 mg/kg and 50 mg/kg i.p.) 30 min. before ALI	2 weeks or 24 h	Chronic (0.1 g/l or 0.25 g/l) and acute caffeine (50 mg/kg i.p.) attenuated the lung edema, hemorrhage, neutrophil recruitment, TNFα and IL-1 expressions, increased lung cAMP and upregulation of A2B receptor mRNAs

**Table 3:** Clinical trials of PDE inhibitors use in patients with ARDS or sepsis.

Author (Year)	Diagnosis	Trial Design	Total No. of Patients	Treatment/Dose	Treatment Duration	Outcomes in the Treated Groups
Salari et al. (2005)	Early ARDS	Prospective clinical investigation	30 (30 PDEI/0 placebo)	Aminophylline (NS-PDE-I) (3 mg/kg i.v. over 30 min., then 15 mg/h)	8 h	Improvement in oxygenation and APACHE score, decrease in serum level of epidermal growth factor (EGF)
Bacher et al. (1997)	Sepsis	Prospective clinical investigation	19 critically ill patients (12 septic, 7 non-septic, both groups treated by PDEI)	Pentoxifylline (NS-PDE-I) (5 mg/kg i.v.)	3 h	In septic patients, increases in heart rate and cardiac index, decrease in systemic vascular resistance index and pulmonary vascular resistance index. No hemodynamic changes in non-septic patients. In both groups, increased oxygen transport (DO_2_) and oxygen uptake (VO_2_), in unchanged oxygen extraction ratio.
Maldonado et al. (2021)	COVID-19-induced moderate to severe ARDS	External pilot study	38 (26 treated by PDEI, 12 controls)	Pentoxifylline (NS-PDE-I) (400 mg p.o. every 8 h)	from admission to discharge	Increased lymphocyte count, decreased serum LDH, a trend towards reduced hospitalization days, mortality, and proportion of patients requiring intubation.
The ARDS Network 2002	ARDS	Prospective, randomized, placebo-controlled, multicenter study	235 (116 treated by PDEI, 119 placebo)	Lisofylline (NS-PDE-I) (3 mg/kg with a maximum dose of 300 mg i.v. every 6 h)	20 days or 48 h after unassisted breathing	Because of no evidence of beneficial effects, the trial was stopped.
Barton et al. (1996)	Pediatric non-hyperdynamic septic shock	Prospective, randomized, placebo-controlled, descriptive, interventional study.	12 (12 treated by milrinone)	Milrinone (PDE3-I) (50 μg/kg i.v. followed by 0.5 μg/kg/min) or placebo for 2 h, then switch for next 2 h	4 h	Increased cardiac index, stroke volume index, right and left ventricular stroke work index, and DO_2_. Decreased systemic vascular resistance index, pulmonary vascular resistance index, and mean pulmonary arterial pressure.
Wang et al. (2015)	Severe sepsis	Prospective randomized study	90 (30 milrinone, 30 milrinone + β-blocker esmolol), 30 placebo)	Milrinone (PDE3-I) (30 μg/kg i.v., maintained at 0.375–0.5 μg/kg/min by infusion	96 h resp. 28 days	Milrinone plus esmolol improved cardiac function and 28-day survival rates, reduced heart rate, and inhibited inflammatory response.
Cornet et al. (2010)	Early ARDS (≤1 week from diagnosis), adults	Prospective, open-label, multicenter, interventional cohort study	10 (10 PDEI/0 placebo)	Sildenafil citrate (PDE5-I) (single dose of 50 mg, via nasogastric tube)	1 day	Decrease in pulmonary artery pressure, systemic mean arterial pressure, no improvement in oxygenation, increased shunt fraction.

**Table 4:** Studies of post-treatment with selective PDE inhibitors in animal models of ARDS or sepsis.

Author (Year)	Type of Injury	Species of Animals	Treatment/Dose	Treatment Duration	Outcomes in Treated Groups
Kuniyoshi et al. (2005)	LPS-induced endotoxemia (LPS infusion, 1.7 mg/kg/h)	Male pigs	Olprinone (PDE3-I) (0.3 mg/kg/min i.v.), given 150 to 240 min from LPS infusion	1.5 h	Increased hepatic oxygen delivery and consumption, prevented a decrease in cytochrome aa3 and an increase in lactate
Mazzon et al. (2011)	Zymosan-induced septic shock (zymosan 500 mg/kg i.p.)	Male CD mice	Olprinone (PDE3-I) (0.2 mg/kg i.p.) at 1 and 6 h after zymosan)	17 h/ 7 days	Decreased peritoneal exudation and migration of PMNs, reduced lung, liver, renal and pancreatic injury, diminished pro-inflammatory markers in tissues, prevented systemic toxicity, loss in body weight, and mortality
Oishi et al. (2012)	Cecal ligation and puncture (CLP) model of sepsis	Male BALB/c mice	Olprinone (PDE3-I) (2 μg/kg i.p.), given immediately after CLP	24 h	Enhanced oxygenation, reduced pulmonary vascular permeability, lung damage, and lung cell apoptosis, improved survival
Mokra et al. (2012)	Meconium-induced ALI	Adult rabbits	Olprinone (PDE3-I) (0.2 mg/kg i.v. bolus, given 30 min. after meconium)	5 h	Decreased PMNs in BALF, lung edema formation, and oxidation markers, prevented a decrease in lung TAS, decreased TBARS and increased TAS in the plasma
Kosutova et al. (2020)	Saline lung lavage-induced ALI	Adult rabbits	Olprinone (PDE3-I) (1 mg/kg i.v. bolus, given 15 min. after the last lung lavage)	4 h	Reduced lung PMNs and edema formation, improved oxygenation, reduced right-to-left pulmonary shunts, decreased markers of lung inflammation and oxidative stress, and apoptosis of epithelial cells
Shao et al. (2019)	Meconium-induced ALI	Newborn piglets	Milrinone (PDE3-I) (50 μg/kg i.v., followed by infusion of 0.75 μg/kg/h) Rolipram (PDE4-I) (0.8 mg/kg i.v.)	4 h	Improved oxygenation, tended to decrease markers of inflammation (milrinone: IL-1β and IL-6 in BALF; rolipram: IL-1β in serum, IL-1β, IL-6 in BALF, CRP in lung).
Özer et al. (2020)	CLP-induced model of sepsis	Wistar rats	Milrinone (PDE3-I) (1/mg/kg/day i.p.) for 72 h after CLP Sildenafil (PDE5-I) (10/mg/kg/day i.p.) for 72 hours after CLP	7 days	Increased antioxidant enzymes in lung and kidney, lowered pathological score in lung and kidney
Holthoff et al. (2013)	Cecal ligation and puncture (CLP) model of sepsis	Male C57BL/6 mice	Rolipram (PDE4-I) (doses of 0.3-10 mg/kg i.p., given 6 h after CLP)	6 or 18 h	Increased renal blood flow and glomerular filtration rate, reduced renal microvascular permeability, blood urea nitrogen, and serum creatinine.
Flemming et al. (2014)	Colon ascendens stent peritonitis (CASP) model of sepsis	Male Sprague-Dawley rats	Rolipram (PDE4-I) (low-dose 1 mg/kg/h vs high-dose 3 mg/kg/h i.v., given 12 h after operation)	14 h	Reduced albumin extravasation and improved mesenteric microcirculatory flow at low-dose, but adverse effects at high-dose. Prevented sepsis-induced acute kidney injury, decreased lung edema and neutrophil infiltration, enhanced oxygenation, decreased IL-1α, IL-1β, IL-12, and TNFα
Wollborn et al. (2015)	LPS-induced acute endotoxemia(LPS 2.5 mg/kg i.v.)	Male Sprague-Dawley rats	Rolipram (PDE4-I) (300 μg/100 g i.v., given 1 h after LPS)	4 h	Improved hepatic volumetric flow and cell death; no influence on inflammatory impact
Sims et al. (2017)	Cecal ligation and puncture (CLP) model of sepsis	Rat pups (17-18-days old)	Rolipram (PDE4-I) (0.1 mg/kg i.p., single dose at 6 h post-CLP)	6 or 18 h/4 days	Restored cardiac function and renal microcirculation, reduced microvascular leakage, improved 4-day survival
Schick et al. (2012)	LPS-induced acute endotoxemia(LPS 5 and 2.5 mg/kg i.v.)	Male Sprague-Dawley rats	Rolipram (PDE4-I) (3 mg/kg i.v. followed by 0.04 μg/100 g/h) Roflumilast (PDE4-I) (240 μg/kg i.v. followed by 4 μg/100 g/h or by repeated boli 240 μg/kg), with or 1.5 h after LPS)	6 h	Both PDE4-I reduced albumin extravasation, stabilized endothelial barrier, and increased microcirculatory flow in mesenteric venules, reduced mortality, no serious adverse effects
Konrad et al. (2015)	Nebulized LPS-induced model of ALI (total of 7 ml, 500 μg/ml)	C57BL/6 male mice	Rolipram (PDE4-I) (1mg/kg i.p.), Roflumilast (PDE4-I) (500 μg/kg i.p.), given 1h after LPS inhalation	6 h	Both PDE4-I decreased cell migration and TNFα, IL6, CXCL1 and CXCL2/3, both PDE4-I decreased LPS-induced PDE4B
Kosutova et al. (2017)	Saline-lavage induced surfactant depletion	New Zealand rabbits	Roflumilast (PDE4-I) (1 mg/kg i.v.)	4 h	Reduced leak of neutrophils into the lung and lung edema formation, improved respiratory parameters
Kosutova et al. (2018a)	Saline-lavage induced surfactant depletion	New Zealand rabbits	Roflumilast (PDE4-I) (1 mg/kg i.v.)	4 h	Reduced lung edema generation, apoptosis of lung epithelial cells, and neutrophil count in BALF, decreased TNFα, IL-6, IL-8, MDA in the lung and plasma, and 3-nitrotyrosine in the lung
Shekerdemian et al. (2002)	Meconium-induced ALI	Piglets	Sildenafil (PDE5-I) (2 mg/kg i.v. infusion over 2 h) started at 2 h after meconium	2 h	Reversed an increase in pulmonary vascular resistance, no effect on systemic hemodynamics. Increased cardiac output by 30%, but no influence on oxygenation
Ryhammer et al. (2006)	Meconium-induced ALI	Piglets	Sildenafil (PDE5-I) (hourly an increasing dose 0.4, 1, and 3 mg/kg i.v., starting 2 h after meconium)	3 h	Reduced pulmonary artery pressure and pulmonary vascular resistance by 30%, but increased oxygenation index
Cadirci et al. (2011)	Cecal ligation and puncture (CLP) model of sepsis	Male Wistar rats	Sildenafil (PDE5-I) (10 or 20 mg/kg p.o., given immediately after CLP)	16 h	Increased GSH levels, decreased levels of SOD and activation of MPO and LPO; at higher dose: improved biochemical status of lung and kidneys in the sham-operated rats, improved inflammation scores
Gokakin et al. (2013)	Severe scald burn-induced ALI	Female Wistar rats	Sildenafil (PDE5-I) (10 or 20 mg/kg p.o., given immediately after the scald burn	24 h	Decreased lung inflammation scores and oxidation markers, increased anti-oxidation markers
Kovalski et al. (2017)	CLP-induced sepsis	Male Wistar rats	Sildenafil (PDE5-I) (10 mg/kg, gavage, given 8 h after the insult	16 h	Increased renal blood flow and reduced plasma levels of creatinine, lactate and creatine kinase, and reduced lung MPO
Kosutova et al. (2018b)	Saline-lavage induced surfactant depletion	New Zealand rabbits	Sildenafil (PDE5-I) (1 mg/kg i.v.)	4 h	Reduced neutrophils in BALF, TNFα, IL-6, IL-8, nitrite/nitrate, MDA, and 3-nitrotyrosine in the lung, reduced lung edema and apoptosis of lung epithelial cells
Fang et al. (2020)	Severe acute pancreatitis-induced ALI	Male SD rats	Sildenafil (PDE5-I) (100 mg/kg i.p.) given 2 h after the insult	10 h	Prevented ALI and neutrophil infiltration, decreased IL-1β, IL-6 and TNFα, promoted proliferation and inhibited apoptosis, inhibited NF-kB signal pathway
Rocco et al. (2003)	LPS-induced ALI (10 μg i.t.)	BALB/c mice	LASSBio596 (PDE4/5-I) (10 mg/kg i.p., given 1 h before or 6 h after LPS)	24 h	Enhanced lung mechanics, inhibited neutrophil recruitment and TNFα release, prevented LPS- induced bronchoconstriction, alveolar collapse, and increase of collagen
Świerczek et al. (2020)	LPS-induced endotoxemia (1 mg/kg i.v.)	Wistar rats	GRMS-55 (PDE7-I) (20 mg/kg i.v., with LPS)	3 h	Decreased plasma TNFα

## References

[1] Bernard G.R., Artigas A., Brigham K.L., Carlet J., Falke K., Hudson L., Lamy M., Legall J.R., Morris A., Spragg R. (1994). The American-European Consensus Conference on ARDS. Definitions, mechanisms, relevant outcomes, and clinical trial The American-European Consensus Conference on ARDS. Definitions, mechanisms, relevant outcomes, and clinical trial coordi-nation. Am. J. Respir. Crit. Care Med..

[2] Ranieri V.M., Rubenfeld G.D., Thompson B.T., Ferguson N.D., Caldwell E., Fan E., Campo-rota L., Slutsky A.S., ARDS Definition Task Force (2012). Acute respiratory distress syndrome: The Berlin Definition. JAMA.

[3] Seeley E.J. (2013). Updates in the Management of Acute Lung Injury: A Focus on the Overlap Between AKI and ARDS. Adv. Chronic Kidney Dis..

[4] Rubenfeld G.D., Caldwell E., Peabody E., Weaver J., Martin D.P., Neff M., Stern E.J., Hudson L.D. (2005). Incidence and Outcomes of Acute Lung Injury. New Engl. J. Med..

[5] Bellani G., Laffey J.G., Pham T., Fan E., Brochard L., Esteban A., Gattinoni L., Van Haren F., Larsson A., McAuley D.F. (2016). Epidemiology, Patterns of Care, and Mortality for Patients With Acute Respiratory Distress Syndrome in Intensive Care Units in 50 Countries. JAMA.

[6] Herridge M.S., Moss M., Hough C.L., Hopkins R.O., Rice T.W., Bienvenu O.J., Azoulay E. (2016). Recovery and outcomes after the acute respiratory distress syndrome (ARDS) in patients and their family caregivers. Intensiv. Care Med..

[7] Bein T., Weber-Carstens S., Apfelbacher C. (2018). Long-term outcome after the acute respiratory distress syndrome: Different from general critical illness?. Curr. Opin. Crit. Care..

[8] Ware L.B., Matthay M.A. (2000). The acute respiratory distress syndrome. N. Engl. J. Med..

[9] Liu C., Jg L. (2014). Role of genetic factors in the development of acute respiratory distress syndrome. J. Transl. Intern. Med..

[10] Hernández-Beeftink T., Guillen-Guio B., Villar J., Flores C. (2019). Genomics and the Acute Respiratory Distress Syndrome: Current and Future Directions. Int. J. Mol. Sci..

[11] Lynn H., Sun X., Casanova N., Gonzales-Garay M., Bime C., Garcia J.G.N. (2019). Genomic and Genetic Approaches to Deci-phering Acute Respiratory Distress Syndrome Risk and Mortality. Antioxid. Redox. Signal..

[12] Bime C., Poongkunran C., Borgstrom M., Natt B., Desai H., Parthasarathy S., Garcia J.G.N. (2016). Racial Differences in Mortality from Severe Acute Respiratory Failure in the United States, 2008–2012. Ann. Am. Thorac. Soc..

[13] Reilly J.P., Meyer N.J., Shashaty M.G.S., Feng R., Lanken P.N., Gallop R., Kaplan S., Herlim M., Oz N.L., Hiciano I. (2014). ABO Blood Type A Is Associated With Increased Risk of ARDS in Whites Following Both Major Trauma and Severe Sepsis. Chest.

[14] Matthay M.A., Ware L.B., Zimmerman G.A. (2012). The acute respiratory distress syndrome. J. Clin. Invest..

[15] Spadaro S., Park M., Turrini C., Tunstall T., Thwaites R., Mauri T., Ragazzi R., Ruggeri P., Hansel T.T., Caramori G. (2019). Biomarkers for Acute Respiratory Distress syndrome and prospects for personalised medicine. J. Inflamm..

[16] Mokra D., Kosutova P. (2015). Biomarkers in acute lung injury. Respir. Physiol. Neurobiol..

[17] Blondonnet R., Constantin J.-M., Sapin V., Jabaudon M. (2016). A Pathophysiologic Approach to Biomarkers in Acute Respiratory Distress Syndrome. Dis. Markers.

[18] Galani V., Tatsaki E., Bai M., Kitsoulis P., Lekka M., Nakos G., Kanavaros P. (2010). The role of apoptosis in the pathophysiology of Acute Respiratory Distress Syndrome (ARDS): An up-to-date cell-specific review. Pathol. Res. Pr..

[19] Cross L.M., Matthay M.A. (2011). Biomarkers in Acute Lung Injury: Insights into the Pathogenesis of Acute Lung Injury. Crit. Care Clin..

[20] Umbrello M., Formenti P., Bolgiaghi L., Chiumello D. (2016). Current Concepts of ARDS: A Narrative Review. Int. J. Mol. Sci..

[21] Fan E., Del Sorbo L., Goligher E.C., Hodgson C.L., Munshi L., Walkey A.J., Adhikari N.K., Amato M.B., Branson R., Brower R.G. (2017). An Official American Thoracic Society/European Society of Intensive Care Medicine/Society of Critical Care Medicine Clinical Practice Guideline: Mechanical Ventilation in Adult Patients with Acute Respiratory Distress Syndrome. Am. J. Respir. Crit. Care Med..

[22] Chiumello D., Algieri I., Grasso S., Terragni P., Pelosi P. (2015). Recruitment maneuvers in acute respiratory distress syndrome and during general anesthesia. Minerva Anestesiol.

[23] Lichtenstein D.A., Mezière G.A. (2008). Relevance of Lung Ultrasound in the Diagnosis of Acute Respiratory Failure*: The BLUE Protocol. Chest.

[24] Guérin C., Mancebo J. (2015). Prone positioning and neuromuscular blocking agents are part of standard care in severe ARDS pa-tients: Yes. Intensive Care Med..

[25] Boyle A.J., Mac Sweeney R., McAuley D.F. (2013). Pharmacological treatments in ARDS; A state-of-the-art update. BMC Med..

[26] Standiford T.J., Ward P.A. (2016). Therapeutic targeting of acute lung injury and acute respiratory distress syndrome. Transl. Res..

[27] Neto A.S., Pereira V.G.M., Espósito D.C., Damasceno M.C.T., Schultz M.J. (2012). Neuromuscular blocking agents in patients with acute respiratory distress syndrome: A summary of the current evidence from three randomized controlled trials. Ann. Intensiv. Care.

[28] Tongyoo S., Permpikul C., Mongkolpun W., Vattanavanit V., Udompanturak S., Kocak M., Meduri G.U. (2016). Hydrocortisone treatment in early sepsis-associated acute respiratory distress syndrome: Results of a randomized controlled trial. Crit. Care.

[29] Meduri G.U., Siemieniuk R.A.C., Ness R.A., Seyler S.J. (2018). Prolonged low-dose methylprednisolone treatment is highly effective in reducing duration of mechanical ventilation and mortality in patients with ARDS. J. Intensiv. Care.

[30] Mokra D., Mikolka P., Kosutova P., Mokry J. (2019). Corticosteroids in Acute Lung Injury: The Dilemma Continues. Int. J. Mol. Sci..

[31] Lugnier C. (2006). Cyclic nucleotide phosphodiesterase (PDE) superfamily: A new target for the development of specific therapeutic agents. Pharmacol. Ther..

[32] Francis S.H., Blount M.A., Corbin J.D. (2011). Mammalian cyclic nucleotide phosphodiesterases: Molecular mechanisms and physi-ological functions. Physiol. Rev..

[33] Giembycz M.A., Maurice D.H. (2014). Cyclic nucleotide-based therapeutics for chronic obstructive pulmonary disease. Curr. Opin. Pharmacol..

[34] Ghosh R., Sawant O., Ganpathy P., Pitre S., Kadam V.J. (2009). Phosphodiesterase inhibitors: Their role and implications. Int. J. Pharm.Tech. Res..

[35] Singh N., Shreshtha A.K., Thakur M., Patra S. (2018). Xanthine scaffold: Scope and potential in drug development. Heliyon.

[36] Spina D., Page C.P. (2016). Xanthines and Phosphodiesterase Inhibitors. Handb. Exp. Pharmacol..

[37] Barnes P.J. (2005). Theophylline in Chronic Obstructive Pulmonary Disease: New Horizons. Proc. Am. Thorac. Soc..

[38] Bender A.T., Beavo J.A. (2006). Cyclic Nucleotide Phosphodiesterases: Molecular Regulation to Clinical Use. Pharmacol. Rev..

[39] Joskova M., Mokry J., Franova S. (2020). Respiratory Cilia as a Therapeutic Target of Phosphodiesterase Inhibitors. Front. Pharmacol..

[40] Lapenna D., De Gioia S., Mezzetti A., Ciofani G., Festi D., Cuccurullo F. (1995). Aminophylline: Could it act as an antioxidant in vivo?. Eur. J. Clin. Investig..

[41] Broderick C., Forster R., Abdel-Hadi M., Salhiyyah K. (2020). Pentoxifylline for intermittent claudication. Cochrane Database Syst. Rev..

[42] Parker R., Armstrong M.J., Corbett C., Rowe I.A., Houlihan D.D. (2013). Systematic review: Pentoxifylline for the treatment of severe alcoholic hepatitis. Aliment. Pharmacol. Ther..

[43] DiNicolantonio J.J., Barroso-Aranda J. (2020). Harnessing adenosine A2A receptors as a strategy for suppressing the lung inflam-mation and thrombotic complications of COVID-19: Potential of pentoxifylline and dipyridamole. Med. Hypotheses.

[44] Guidot D.M., Bursten S.L., Rice G.C., Chaney R.B., Singer J.W., Repine A.J., Hybertson B.M., Repine J.E. (1997). Modulating phosphatidic acid metabolism decreases oxidative injury in rat lungs. Am. J. Physiol. Content.

[45] Chen J., Jin L., Chen X. (2018). Efficacy and Safety of Different Maintenance Doses of Caffeine Citrate for Treatment of Apnea in Premature Infants: A Systematic Review and Meta-Analysis. BioMed Res. Int..

[46] Lodha A., Seshia M., McMillan D.D., Barrington K., Yang J., Lee S.K., Shah P.S. (2015). Association of Early Caffeine Administration and Neonatal Outcomes in Very Preterm Neonates. JAMA Pediatr..

[47] Sweet D.G., Carnielli V., Greisen G., Hallman M., Ozek E., Plavka R., Saugstad O.D., Simeoni U., Speer C.P., Vento M. (2017). European Consensus Guidelines on the Management of Respiratory Distress Syndrome - 2016 Update. Neonatology.

[48] Geraets L., Haegens A., Weseler A.R., Brauers K., Vernooy J.H., Wouters E.F., Bast A., Hageman G.J. (2010). Inhibition of acute pulmonary and systemic inflammation by 1,7-dimethylxanthine. Eur. J. Pharmacol..

[49] Escofier N., Boichot E., Germain N., Silva P.M., Martins M.A., Lagente V. (1999). Effects of interleukin-10 and modulators of cyclic AMP formation on endotoxin-induced inflammation in rat lung. Fundam. Clin. Pharmacol..

[50] Mizus I., Summer W., Farrukh I., Michael J.R., Gurtner G.H. (1985). Isoproterenol or aminophylline attenuate pulmonary edema after acid lung injury. Am. Rev. Respir. Dis..

[51] Hsu K., Wang D., Chang M.L., Wu C.P., Chen H.I. (1996). Pulmonary edema induced by phorbol myristate acetate is attenuated by compounds that increase intracellular cAMP. Res. Exp. Med. (Berl)..

[52] Harada H., Ishizaka A., Yonemaru M., Mallick A.A., Hatherill J.R., Zheng H., Lilly C.M., O’Hanley P.T., Raffin T.A. (1989). The Effects of Aminophylline and Pentoxifylline on Multiple Organ Damage afterEscherichia coliSepsis. Am. Rev. Respir. Dis..

[53] Sciuto A.M., Strickland P.T., Kennedy T.P., Gurtner G.H. (1997). Postexposure Treatment with Aminophylline Protects Against Phosgene-Induced Acute Lung Injury. Exp. Lung Res..

[54] Fakioglu H., Gelvez J., Torbati D., Glover M.L., Olarte J.L., Camacho M.T., Wolfsdorf J. (2004). Aminophylline therapy during endotoxemia in anesthetized spontaneously breathing rats. Pharmacol. Res..

[55] Mokra D., Mokry J., Tatarkova Z., Redfors B., Petraskova M., Calkovska A. (2007). Aminophylline treatment in meconium-induced acute lung injury in a rabbit model. J. Physiol. Pharmacol. Off. J. Pol. Physiol. Soc..

[56] Mokra D., Drgova A., Mokry J., Pullmann R., Redfors B., Petraskova M., Calkovska A. (2008). Comparison of the effects of low-dose vs. high-dose aminophylline on lung function in experimental meconium aspiration syndrome. J. Physiol. Pharmacol. Off. J. Pol. Physiol. Soc..

[57] Shao J.-I., Lin C.-H., Yang Y.-H., Jeng M.-J. (2019). Effects of intravenous phosphodiesterase inhibitors and corticosteroids on severe meconium aspiration syndrome. J. Chin. Med Assoc..

[58] Lechner A.J., Rouben L.R., Potthoff L.H., Tredway T.L., Matuschak G.M. (1993). Effects of pentoxifylline on tumor necrosis factor production and survival during lethal E. coli sepsis vs. disseminated candidiasis with fungal septic shock. Circ. Shock..

[59] Oliveira-Junior I., Brunialti M., Koh I., Junqueira V., Salomão R. (2006). Effect of pentoxifylline on lung inflammation and gas exchange in a sepsis-induced acute lung injury model. Braz. J. Med Biol. Res..

[60] Turhan A.H., Atıcı A., Muşlu N., Polat A., Helvacı I. (2012). The effects of pentoxifylline on lung inflammation in a rat model of meconium aspiration syndrome. Exp. Lung Res..

[61] Li Q., Hu X., Sun R., Tu Y., Gong F., Ni Y. (2016). Resolution acute respiratory distress syndrome through reversing the imbalance of Treg/Th17 by targeting the cAMP signaling pathway. Mol. Med. Rep..

[62] Hoffmann H., Hatherill J.R., Crowley J., Harada H., Yonemaru M., Zheng H., Ishizaka A., Raffin T.A. (1991). Early Post-Treatment with Pentoxifylline or Dibutyryl cAMP AttenuatesEscherichia coli-induced Acute Lung Injury in Guinea Pigs. Am. Rev. Respir. Dis..

[63] Ridings P.C., Windsor A.C.J., Sugerman H.J., Kennedy E., Sholley M.M., Blocher C.R., Fisher B.J., Fowler A.A. (1994). Beneficial Cardiopulmonary Effects of Pentoxifylline in Experimental Sepsis Are Lost Once Septic Shock Is Established. Arch. Surg..

[64] Nelson J.L., Alexander J.W., Mao J.X., Vohs T., Ogle C.K. (1999). Effect of pentoxifylline on survival and intestinal cytokine mes-senger RNA transcription in a rat model of ongoing peritoneal sepsis. Crit. Care Med..

[65] Ji Q., Zhang L., Jia H., Xu J. (2004). Pentoxifylline inhibits endotoxin-induced NF-kappa B activation and associated production of proinflammatory cytokines. Ann. Clin. Lab. Sci..

[66] Coimbra R., Melbostad H., Loomis W., Tobar M., Hoyt D.B. (2005). Phosphodiesterase inhibition decreases nuclear factor-kappaB activation and shifts the cytokine response toward anti-inflammatory activity in acute endotoxemia. J. Trauma..

[67] Coimbra R., Melbostad H., Loomis W., Wolf P., Tobar M., Hoyt D.B. (2006). LPS-Induced Acute Lung Injury is Attenuated by Phosphodiesterase Inhibition: Effects on Proinflammatory Mediators, Metalloproteinases, NF-??B, and ICAM-1 Expression. J. Trauma Inj. Infect. Crit. Care.

[68] Özer Ö., Topal U., Şen M. (2020). The effects of specific and non-specific phosphodiesterase inhibitors and N-acetylcysteine on oxidative stress and remote organ injury in two-hit trauma model. Ulus. Travma. Acil. Cerrahi. Derg..

[69] Korhonen K., Kiuru A., Svedström E., Kääpä P. (2004). Pentoxifylline reduces regional inflammatory and ventilatory disturbances in meconium-exposed piglet lungs. Pediatr. Res..

[70] Sunil V.R., Vayas K.N., Cervelli J.A., Malaviya R., Hall L., Massa C.B., Gow A.J., Laskin J.D., Laskin D.L. (2014). Pentoxifylline attenuates nitrogen mustard-induced acute lung injury, oxidative stress and inflammation. Exp. Mol. Pathol..

[71] Hybertson B.M., Bursten S.L., Leff J.A., Lee Y.M., Jepson E.K., DeWitt C.R., Zagorski J., Cho H.G., Repine J.E. (1997). Lisofylline prevents leak, but not neutrophil accumulation, in lungs of rats given IL-1 intratracheally. J. Appl. Physiol..

[72] Hasegawa N., Oka Y., Nakayama M., Berry G.J., Bursten S., Rice G., Raffin T.A. (1997). The effects of post-treatment with li-sofylline, a phosphatidic acid generation inhibitor, on sepsis-induced acute lung injury in pigs. Am. J. Respir. Crit. Care Med..

[73] Oka Y., Hasegawa N., Nakayama M., Murphy G.A., Sussman H.H., Raffin T.A. (1999). Selective Downregulation of Neutrophils by a Phosphatidic Acid Generation Inhibitor in a Porcine Sepsis Model. J. Surg. Res..

[74] Weichelt U., Cay R., Schmitz T., Strauss E., Sifringer M., Bührer C., Endesfelder S. (2012). Prevention of hyperoxia-mediated pulmonary inflammation in neonatal rats by caffeine. Eur. Respir. J..

[75] Jing X., Huang Y.-W., Jarzembowski J., Shi Y., Konduri G.G., Teng R.-J. (2017). Caffeine ameliorates hyperoxia-induced lung injury by protecting GCH1 function in neonatal rat pups. Pediatr. Res..

[76] Teng R.-J., Jing X., Michalkiewicz T., Afolayan A.J., Wu T.-J., Konduri G.G. (2017). Attenuation of endoplasmic reticulum stress by caffeine ameliorates hyperoxia-induced lung injury. Am. J. Physiol. Cell. Mol. Physiol..

[77] Endesfelder S., Weichelt U., Strauß E., Schlör A., Sifringer M., Scheuer T., Bührer C., Schmitz T. (2017). Neuroprotection by Caffeine in Hyperoxia-Induced Neonatal Brain Injury. Int. J. Mol. Sci..

[78] Li J., Li G., Hu J.-L., Fu X.-H., Zeng Y.-J., Zhou Y.-G., Xiong G., Yang N., Dai S.-S., He F.-T. (2011). Chronic or high dose acute caffeine treatment protects mice against oleic acid-induced acute lung injury via an adenosine A2A receptor-independent mechanism. Eur. J. Pharmacol..

[79] Fehrholz M., Speer C.P., Kunzmann S. (2014). Caffeine and Rolipram Affect Smad Signalling and TGF-β1 Stimulated CTGF and Transgelin Expression in Lung Epithelial Cells. PLoS ONE.

[80] Salari P., Mojtahedzadeh M., Najafi A., Sadraie S., Bahaadini K., Moharreri M., Hadavand N., Abdollahi M. (2005). Comparison of the effect of aminophylline and low PEEP vs. high PEEP on EGF concentration in critically ill patients with ALI/ARDS. J. Clin. Pharm. Ther..

[81] Bacher A., Mayer N., Klimscha W., Oismuller C., Steltzer H., Hammerle A. (1997). Effects of pentoxifylline on hemodynamics and oxygenation in septic and nonseptic patients. Crit. Care Med..

[82] Pammi M., Haque K.N. (2015). Pentoxifylline for treatment of sepsis and necrotizing enterocolitis in neonates. Cochrane Database Syst. Rev..

[83] Shabaan A.E., Nasef N., Shouman B., Nour I., Mesbah A., Abdel-Hady H. (2015). Pentoxifylline therapy for late-onset sepsis in preterm infants: A randomized controlled trial. Pediatr. Infect. Dis. J..

[84] Tian J., Shen P., Pan K., Zhou Q. (2019). Efficacy of pentoxifylline treatment for neonatal sepsis: A meta-analysis of randomized controlled studies. Ital. J. Pediatr..

[85] Kaempfen S., Patole S.K. (2014). Pentoxifylline for the prevention of bronchopulmonary dysplasia in preterm infants. Cochrane Database Syst. Rev..

[86] Harris E., Schulzke S.M., Patole S.K. (2010). Pentoxifylline in preterm neonates: A systematic review. Paediatr. Drugs..

[87] Speer E.M., Dowling D.J., Ozog L.S., Xu J., Yang J., Kennady G., Levy O. (2017). Pentoxifylline inhibits TLR- and inflam-masome-mediated in vitro inflammatory cytokine production in human blood with greater efficacy and potency in newborns. Pediatr. Res..

[88] Schüller S.S., Wisgrill L., Herndl E., Spittler A., Förster-Waldl E., Sadeghi K., Kramer B.W., Berger A. (2017). Pentoxifylline modulates LPS-induced hyperinflammation in monocytes of preterm infants in vitro. Pediatr. Res..

[89] Martin J.F.B., Jimenez J.L., Muńoz-Fernández A. (2003). Pentoxifylline and severe acute respiratory syndrome (SARS): A drug to be considered. Med Sci. Monit..

[90] Adhikari N.K., Burns K.E., Meade O.M., Ratnapalan M. (2004). Pharmacologic therapies for adults with acute lung injury and acute respiratory distress syndrome. Cochrane Database Syst. Rev..

[91] Bursten S.L., Federighi D.A., Parsons P., Harris W.E., Abraham E., Moore E.E., Moore F.A., Bianco J.A., Singer J.W., Repine J.E. (1996). An increase in serum C18 unsaturated free fatty acids as a predictor of the development of acute respiratory distress syndrome. Crit. Care Med..

[92] Network T.A.C.T. (2002). National Institutes of Health Randomized, placebo-controlled trial of lisofylline for early treatment of acute lung injury and acute respiratory distress syndrome. Crit. Care Med..

[93] Hashimoto S., Sanui M., Egi M., Ohshimo S., Shiotsuka J., Seo R., Tanaka R., Tanaka Y., Norisue Y., Hayashi Y. (2017). ARDS clinical practice guideline committee from the Japanese Society of Respiratory Care Medicine and the Japanese Society of Intensive Care Medicine. The clinical practice guideline for the management of ARDS in Japan. J. Intensive Care..

[94] Zhang Y.-S., Li J.-D., Yan C. (2018). An update on vinpocetine: New discoveries and clinical implications. Eur. J. Pharmacol..

[95] O’Brien J.J., O’Callaghan J.P., Miller D.B., Chalgeri S., Wennogle L.P., Davis R.E., Snyder G.L., Hendrick J.P. (2020). Inhibition of calcium-calmodulin-dependent phosphodiesterase (PDE1) suppresses inflammatory responses. Mol. Cell. Neurosci..

[96] Wennogle L.P., Hoxie H., Peng Y., Hendrick J.P. (2017). Phosphodiesterase 1: A Unique Drug Target for Degenerative Diseases and Cognitive Dysfunction. Adv. Neurobiol..

[97] Schermuly R.T., Pullamsetti S.S., Kwapiszewska G., Dumitrascu R., Tian X., Weissmann N., Ghofrani H.A., Kaulen C., Dunkern T., Schudt C. (2007). Phosphodiesterase 1 upregulation in pulmonary arterial hypertension: Target for reverse-remodeling therapy. Circulation.

[98] Chan S., Yan C. (2011). PDE1 isozymes, key regulators of pathological vascular remodeling. Curr. Opin. Pharmacol..

[99] Wu Y., Tian Y.-J., Le M.-L., Zhang S.-R., Zhang C., Huang M.-X., Jiang M.-Y., Zhang B., Luo H.-B. (2020). Discovery of Novel Selective and Orally Bioavailable Phosphodiesterase-1 Inhibitors for the Efficient Treatment of Idiopathic Pulmonary Fibrosis. J. Med. Chem..

[100] Kogiso H., Hosogi S., Ikeuchi Y., Tanaka S., Shimamoto C., Matsumura H., Nakano T., Sano K.I., Inui T., Marunaka Y. (2017). A low [Ca2+]i-induced enhancement of cAMP-activated ciliary beating by PDE1A inhibition in mouse airway cilia. Pflugers Arch..

[101] Mokry J., Nosalova G. (2011). The influence of the PDE inhibitors on cough reflex in guinea pigs. Bratisl Lek List..

[102] Haddad J.J., Land S.C., Tarnow-Mordi W.O., Zembala M., Kowalczyk D., Lauterbach R. (2002). Immunopharmacological Potential of Selective Phosphodiesterase Inhibition. I. Differential Regulation of Lipopolysaccharide-Mediated Proinflammatory Cytokine (Interleukin-6 and Tumor Necrosis Factor-α) Biosynthesis in Alveolar Epithelial Cells. J. Pharmacol. Exp. Ther..

[103] Brown D., Hutchison L., Donaldson K., MacKenzie S., Dick C., Stone V. (2007). The effect of oxidative stress on macrophages and lung epithelial cells: The role of phosphodiesterases 1 and 4. Toxicol. Lett..

[104] Zuo H., Cattani-Cavalieri I., Musheshe N., Nikolaev V.O., Schmidt M. (2019). Phosphodiesterases as therapeutic targets for res-piratory diseases. Pharmacol. Ther..

[105] Sadek M.S., Cachorro E., El-Armouche A., Kämmerer S. (2020). Therapeutic Implications for PDE2 and cGMP/cAMP Mediated Crosstalk in Cardiovascular Diseases. Int. J. Mol. Sci..

[106] Zhang C., Lueptow L.M., Zhang H.T., O’Donnell J.M., Xu Y. (2017). The Role of Phosphodiesterase-2 in Psychiatric and Neuro-degenerative Disorders. Adv. Neurobiol..

[107] Gresele P., Momi S., Falcinelli E. (2011). Anti-platelet therapy: Phosphodiesterase inhibitors. Br. J. Clin. Pharmacol..

[108] Movsesian M., Khan F.A., Hirsch E. (2018). Functions of PDE3 Isoforms in Cardiac Muscle. J. Cardiovasc. Dev. Dis..

[109] Di Paola R., Mazzon E., Paterniti I., Impellizzeri D., Bramanti P., Cuzzocrea S. (2011). Olprinone, a PDE3 inhibitor, modulates the inflammation associated with myocardial ischemia–reperfusion injury in rats. Eur. J. Pharmacol..

[110] Beute J., Lukkes M., Koekoek E.P., Nastiti H., Ganesh K., De Bruijn M.J., Hockman S., Van Nimwegen M., Braunstahl G.-J., Boon L. (2018). A pathophysiological role of PDE3 in allergic airway inflammation. JCI Insight.

[111] Hirota K., Yoshioka H., Kabara S., Kudo T., Ishihara H., Matsuki A. (2001). A Comparison of the Relaxant Effects of Olprinone and Aminophylline on Methacholine-Induced Bronchoconstriction in Dogs. Anesthesia Analg..

[112] Mokry J., Mokra D., Nosalova G., Beharkova M., Feherova Z. (2008). Influence of selective inhibitors of phosphodiesterase 3 and 4 on cough and airway reactivity. J. Physiol. Pharmacol. Off. J. Pol. Physiol. Soc..

[113] Hashimoto Y., Hirota K., Yoshioka H., Kudo T., Ishihara H., Matsuki A. (2000). A comparison of the spasmolytic effects of olprinone and aminophylline on serotonin-induced pulmonary hypertension and bronchoconstriction with or without be-ta-blockade in dogs. Anesth. Analg..

[114] Hashiba E., Hirota K., Yoshioka H., Hashimoto Y., Kudo T., Sato T., Matsuki A. (2000). Milrinone Attenuates Serotonin-Induced Pulmonary Hypertension and Bronchoconstriction in Dogs. Anesthesia Analg..

[115] Milara J., Peiró T., Serrano A., Guijarro R., Zaragozá C., Tenor H., Cortijo J. (2014). Roflumilast N-oxide inhibits bronchial epithelial to mesenchymal transition induced by cigarette smoke in smokers with COPD. Pulm. Pharmacol. Ther..

[116] Sisson T.H., Christensen P.J., Muraki Y., Dils A.J., Chibucos L., Subbotina N., Tohyama K., Horowitz J.C., Matsuo T., Bailie M. (2018). Phosphodiesterase 4 inhibition reduces lung fibrosis following targeted type II alveolar ep-ithelial cell injury. Physiol. Rep..

[117] Mokry J., Urbanova A., Medvedova I., Kertys M., Mikolka P., Kosutova P., Mokra D. (2017). Effects of tadalafil (PDE5 inhibitor) and roflumilast (PDE4 inhibitor) on airway reactivity and markers of inflammation in ovalbumin-induced airway hyperre-sponsiveness in guinea pigs. J. Physiol. Pharmacol..

[118] Milara J., Armengot M., Bañuls P., Tenor H., Beume R., Artigues E., Cortijo J. (2012). Roflumilast N-oxide, a PDE4 inhibitor, improves cilia motility and ciliated human bronchial epithelial cells compromised by cigarette smoke in vitro. Br. J. Pharmacol..

[119] Sebkhi A., Strange J.W., Phillips S.C., Wharton J., Wilkins M.R. (2003). Phosphodiesterase Type 5 as a Target for the Treatment of Hypoxia-Induced Pulmonary Hypertension. Circulation.

[120] Ghofrani H.A., Osterloh I.H., Grimminger F. (2006). Sildenafil: From angina to erectile dysfunction to pulmonary hypertension and beyond. Nat. Rev. Drug Discov..

[121] Urbanova A., Medvedova I., Kertys M., Mikolka P., Kosutova P., Mokra D., Mokrý J. (2017). Dose dependent effects of tadalafil and roflumilast on ovalbumin-induced airway hyperresponsiveness in guinea pigs. Exp. Lung Res..

[122] Laxmi V., Gupta R., Bhattacharya S.K., Ray A., Gulati K. (2019). Inhibitory effects of sildenafil and tadalafil on inflammation, oxidative stress and nitrosative stress in animal model of bronchial asthma. Pharmacol. Rep..

[123] Giembycz M.A., Smith S.J. (2006). Phosphodiesterase 7A: A new therapeutic target for alleviating chronic inflammation?. Curr. Pharm. Des..

[124] Vijayakrishnan L., Rudra S., Eapen M.S., Dastidar S., Ray A. (2007). Small-molecule inhibitors of PDE-IV and -VII in the treatment of respiratory diseases and chronic inflammation. Expert Opin. Investig. Drugs.

[125] Giembycz M.A., Newton R. (2011). Harnessing the Clinical Efficacy of Phosphodiesterase 4 Inhibitors in Inflammatory Lung Diseases: Dual-Selective Phosphodiesterase Inhibitors and Novel Combination Therapies. Handb. Exp. Pharmacol..

[126] Mokrý J., Joskova M., Mokra D., Christensen I., Nosalova G. (2012). Effects of Selective Inhibition of PDE4 and PDE7 on Airway Reactivity and Cough in Healthy and Ovalbumin-Sensitized Guinea Pigs. Adv. Exp. Med. Biol..

[127] Vang A.G., Basole C., Dong H., Nguyen R.K., Housley W., Guernsey L., Adami A.J., Thrall R.S., Clark R.B., Epstein P.M. (2016). Differential Expression and Function of PDE8 and PDE4 in Effector T cells: Implications for PDE8 as a Drug Target in Inflammation. Front. Pharmacol..

[128] Johnstone T.B., Smith K.H., Koziol-White C.J., Li F., Kazarian A.G., Corpuz M.L., Shumyatcher M., Ehlert F.J., Himes B.E., Panettieri R.A. (2018). PDE8 Is Expressed in Human Airway Smooth Muscle and Selectively Regulates cAMP Sig-naling by β2-Adrenergic Receptors and Adenylyl Cyclase 6. Am. J. Respir. Cell. Mol. Biol..

[129] Tajima T., Shinoda T., Urakawa N., Shimizu K., Kaneda T. (2018). Phosphodiesterase 9 (PDE9) regulates bovine tracheal smooth muscle relaxation. J. Vet. Med. Sci..

[130] Jeon K.I., Xu X., Aizawa T., Lim J.H., Jono H., Kwon D.S., Abe J., Berk B.C., Li J.D., Yan C. (2010). Vinpocetine inhibits NF-kappaB-dependent inflammation via an IKK-dependent but PDE-independent mechanism. Proc. Natl. Acad. Sci. USA.

[131] Ruiz-Miyazawa K.W., Pinho-Ribeiro F.A., Zarpelon A.C., Staurengo-Ferrari L., Silva R.L., Alves-Filho J.C., Cunha T.M., Cunha F.Q., Casagrande R., Verri W.A. (2015). Vinpocetine reduces lipopolysaccharide-induced inflammatory pain and neu-trophil recruitment in mice by targeting oxidative stress, cytokines and NF-κB. Chem. Biol. Interact..

[132] Witzenrath M., Gutbier B., Schmeck B., Tenor H., Seybold J., Kuelzer R., Grentzmann G., Hatzelmann A., Van Laak V., Tschernig T. (2009). Phosphodiesterase 2 inhibition diminished acute lung injury in murine pneumococcal pneumonia*. Crit. Care Med..

[133] Bubb K.J., Trinder S.L., Baliga R.S., Patel J., Clapp L.H., MacAllister R.J., Hobbs A.J. (2014). Inhibition of Phosphodiesterase 2 Augments cGMP and cAMP Signaling to Ameliorate Pulmonary Hypertension. Circulation.

[134] Neviere R., Delguste F., Durand A., Inamo J., Boulanger E., Preau S. (2016). Abnormal Mitochondrial cAMP/PKA Signaling Is Involved in Sepsis-Induced Mitochondrial and Myocardial Dysfunction. Int. J. Mol. Sci..

[135] Kuniyoshi T., Kakihana Y., Isowaki S., Nagata E., Tobo K., Kaminosono T., Hashiguchi T., Tahara M., Kawamae H., Okayama N. (2005). Effects of olprinone on hepatosplanchnic circulation and mitochondrial oxidation in a porcine model of endotoxemia. J. Anesthesia.

[136] Kakura H., Miyahara K., Amitani S., Sohara H., Koga M., Sakamoto H., Misumi K., Miura N. (2000). Hemodynamic Effects of Intravenous Administration of Olprinone Hydrochloride on Experimental Pulmonary Hypertension. Arzneimittelforschung.

[137] Koike T., Qutab M.N., Tsuchida M., Takekubo M., Saito M., Hayashi J.-I. (2008). Pretreatment with olprinone hydrochloride, a phosphodiesterase III inhibitor, attenuates lipopolysaccharide-induced lung injury via an anti-inflammatory effect. Pulm. Pharmacol. Ther..

[138] Oishi H., Takano K.-I., Tomita K., Takebe M., Yokoo H., Yamazaki M., Hattori Y. (2012). Olprinone and colforsin daropate alleviate septic lung inflammation and apoptosis through CREB-independent activation of the Akt pathway. Am. J. Physiol. Cell. Mol. Physiol..

[139] Mazzon E., Esposito E., Di Paola R., Impellizzeri D., Bramanti P., Cuzzocrea S. (2011). Olprinone, a specific phosphodiesterase (PDE)-III inhibitor, reduces the development of multiple organ dysfunction syndrome in mice. Pharmacol. Res..

[140] Mokra D., Drgova A., Pullmann R.S., Calkovska A. (2012). Selective phosphodiesterase 3 inhibitor olprinone attenuates meco-nium-induced oxidative lung injury. Pulm. Pharmacol. Ther..

[141] Kosutova P., Mikolka P., Balentova S., Adamkov M., Calkovska A., Mokra D. (2020). Effects of PDE3 Inhibitor Olprinone on the Respiratory Parameters, Inflammation, and Apoptosis in an Experimental Model of Acute Respiratory Distress Syndrome. Int. J. Mol. Sci..

[142] Sanz M.-J., Cortijo J., A Taha M., Cerdá-Nicolás M., Schatton E., Burgbacher B., Klar J., Tenor H., Schudt C., Issekutz A.C. (2007). Roflumilast inhibits leukocyte-endothelial cell interactions, expression of adhesion molecules and microvascular permeability. Br. J. Pharmacol..

[143] Schick M.A., Wunder C., Wollborn J., Roewer N., Waschke J., Germer C.-T., Schlegel N. (2012). Phosphodiesterase-4 inhibition as a therapeutic approach to treat capillary leakage in systemic inflammation. J. Physiol..

[144] Konrad F.M., Bury A., Schick M.A., Ngamsri K.C., Reutershan J. (2015). The unrecognized effects of phosphodiesterase 4 on epi-thelial cells in pulmonary inflammation. PLoS ONE.

[145] Flemming S., Schlegel N., Wunder C., Meir M., Baar W., Wollborn J., Roewer N., Germer C.T., Schick M.A. (2014). Phos-phodiesterase 4 inhibition dose dependently stabilizes microvascular barrier functions and microcirculation in a rodent model of polymicrobial sepsis. Shock.

[146] Selige J., Tenor H., Hatzelmann A., Dunkern T. (2010). Cytokine-dependent balance of mitogenic effects in primary human lung fibroblasts related to cyclic AMP signaling and phosphodiesterase 4 inhibition. J. Cell. Physiol..

[147] Sabatini F., Petecchia L., Boero S., Silvestri M., Klar J., Tenor H., Beume R., Hatzelmann A., Rossi G. (2010). A phosphodiesterase 4 inhibitor, roflumilast N-oxide, inhibits human lung fibroblast functions in vitro. Pulm. Pharmacol. Ther..

[148] Selige J., Hatzelmann A., Dunkern T. (2011). The differential impact of PDE4 subtypes in human lung fibroblasts on cytokine-induced proliferation and myofibroblast conversion. J. Cell. Physiol..

[149] Cortijo J., Iranzo A., Milara X., Mata M., Cerdá-Nicolás M., Ruiz-Saurí A., Tenor H., Hatzelmann A., Morcillo E.J. (2009). Roflumilast, a phosphodiesterase 4 inhibitor, alleviates bleomycin-induced lung injury. Br. J. Pharmacol..

[150] Udalov S., Dumitrascu R., Pullamsetti S.S., Al-Tamari H.M., Weissmann N., A Ghofrani H., Guenther A., Voswinckel R., Seeger W., Grimminger F. (2010). Effects of phosphodiesterase 4 inhibition on bleomycin-induced pulmonary fibrosis in mice. BMC Pulm. Med..

[151] Milara J., Morcillo E.J., Monleon D., Tenor H., Cortijo J. (2015). Roflumilast Prevents the Metabolic Effects of Bleomycin-Induced Fibrosis in a Murine Model. PLoS ONE.

[152] De Visser Y.P., Walther F.J., Laghmani E.H., Van Wijngaarden S., Nieuwland K., Wagenaar G.T.M. (2008). Phosphodiesterase-4 inhibition attenuates pulmonary inflammation in neonatal lung injury. Eur. Respir. J..

[153] Ma H., Shi J., Wang C., Guo L., Gong Y., Li J., Gong Y., Yun F., Zhao H., Li E. (2014). Blockade of PDE4B limits lung vascular permeability and lung inflammation in LPS-induced acute lung injury. Biochem. Biophys. Res. Commun..

[154] Miotla J.M., Teixeira M.M., Hellewell P.G. (1998). Suppression of Acute Lung Injury in Mice by an Inhibitor of Phosphodiesterase Type 4. Am. J. Respir. Cell Mol. Biol..

[155] Spond J., Chapman R., Fine J., Jones H., Kreutner W., Kung T., Minnicozzi M. (2001). Comparison of PDE 4 Inhibitors, Rolipram and SB 207499 (ArifloTM, in a Rat Model of Pulmonary Neutrophilia. Pulm. Pharmacol. Ther..

[156] Nials A.T., Tralau-Stewart C.J., Gascoigne M.H., Ball D.I., Ranshaw L.E., Knowles R.G. (2011). In Vivo Characterization of GSK256066, a High-Affinity Inhaled Phosphodiesterase 4 Inhibitor. J. Pharmacol. Exp. Ther..

[157] Kosutova P., Mikolka P., Kolomaznik M., Rezakova S., Calkovska A., Mokra D. (2017). Effects of roflumilast, a phosphodiesterase-4 inhibitor, on the lung functions in a saline lavage-induced model of acute lung injury. Physiol. Res..

[158] Kosutova P., Mikolka P., Kolomaznik M., Balentova S., Adamkov M., Calkovska A., Mokra D. (2018). Reduction of Lung Inflammation, Oxidative Stress and Apoptosis by the PDE4 Inhibitor Roflumilast in Experimental Model of Acute Lung Injury. Physiol. Res..

[159] Wollborn J., Schlegel N., Schick M.A. (2017). Phosphodiesterase-4-Inhibition zur Therapie der endothelialen Schranken- und Mikrozirkulationsstörung in der Sepsis Phosphodiesterase 4 inhibition for treatment of endothelial barrier and microcirculation disorders in sepsis. Der Anaesthesist.

[160] Turner C.R., Esser K.M., Wheeldon E.B. (1993). Therapeutic intervention in a rat model of ARDS: IV. Phosphodiesterase IV inhibition. Circ. Shock..

[161] Holthoff J.H., Wang Z., Patil N.K., Gokden N., Mayeux P.R. (2013). Rolipram Improves Renal Perfusion and Function during Sepsis in the Mouse. J. Pharmacol. Exp. Ther..

[162] Sims C.R., Singh S.P., Mu S., Gokden N., Zakaria D., Nguyen T.C., Mayeux P.R. (2017). Rolipram Improves Outcome in a Rat Model of Infant Sepsis-Induced Cardiorenal Syndrome. Front. Pharmacol..

[163] Millen J., MacLean M.R., Houslay M.D. (2006). Hypoxia-induced remodelling of PDE4 isoform expression and cAMP handling in human pulmonary artery smooth muscle cells. Eur. J. Cell Biol..

[164] Farrow K.N., Steinhorn R.H. (2011). Phosphodiesterases: Emerging Therapeutic Targets for Neonatal Pulmonary Hypertension. Handb. Exp. Pharmacol..

[165] Blanch L., Albaiceta G.M. (2010). Sildenafil for pulmonary hypertension in ARDS: A new pleasant effect?. Intensiv. Care Med..

[166] Zhao L., Mason N., Morrell N., Kojonazarov B., Sydykov A., Maripov A., Mirrakhimov M., Aldashev A., Wilkins M. (2001). Sildenafil Inhibits Hypoxia-Induced Pulmonary Hypertension. Circulation.

[167] Tessler R.B., Zadinello M., Fiori H., Colvero M., Belik J., Fiori R.M. (2008). Tadalafil improves oxygenation in a model of newborn pulmonary hypertension. Pediatr. Crit. Care Med..

[168] Kosutova P., Mikolka P., Balentova S., Kolomaznik M., Adamkov M., Mokry J., Calkovska A., Mokra D. (2019). Effects of phosphodiesterase 5 inhibitor sildenafil on the respiratory parameters, inflammation and apoptosis in a saline lavage-induced model of acute lung injury. J. Physiol. Pharmacol. Off. J. Pol. Physiol. Soc..

[169] Shekerdemian L.S., Ravn H.B., Penny D.J. (2002). Intravenous Sildenafil Lowers Pulmonary Vascular Resistance in a Model of Neonatal Pulmonary Hypertension. Am. J. Respir. Crit. Care Med..

[170] Ryhammer P.K., Shekerdemian L.S., Penny D.J., Ravn H.B. (2006). Effect of Intravenous Sildenafil on Pulmonary Hemodynamics and Gas Exchange in the Presence and Absence of Acute Lung Injury in Piglets. Pediatr. Res..

[171] Kovalski V., Prestes A.P., Oliveira J.G., Alves G.F., Colarites D.F., Mattos J.E., Sordi R., Vellosa J.C., Fernandes D. (2017). Pro-tective role of cGMP in early sepsis. Eur. J. Pharmacol..

[172] Fang D., Lin Q., Wang C., Zheng C., Li Y., Huang T., Ni F., Wu Z., Chen B., Sun L. (2020). Effects of sildenafil on inflammatory injury of the lung in sodium taurocholate-induced severe acute pancreatitis rats. Int. Immunopharmacol..

[173] Gokakin A.K., Deveci K., Kurt A., Karakus B.C., Duger C., Tuzcu M., Topcu O. (2013). The protective effects of sildenafil in acute lung injury in a rat model of severe scald burn: A biochemical and histopathological study. Burns.

[174] Gokakin A.K., Atabey M., Deveci K., Sancakdar E., Tuzcu M., Duger C., Topcu O. (2014). The effects of Sildenafil in liver and kidney injury in a rat model of severe scald burn: A biochemical and histopathological study. Turk. J. Trauma Emerg. Surg..

[175] Cadirci E., Halici Z., Odabasoglu F., Albayrak A., Karakus E., Unal D., Atalay F., Ferah I., Unal B. (2011). Sildenafil treatment attenuates lung and kidney injury due to overproduction of oxidant activity in a rat model of sepsis: A biochemical and his-topathological study. Clin. Exp. Immunol..

[176] Rocco P., Momesso D., Figueira R., Ferreira H., Cadete R., Légora-Machado A., Koatz V., Lima L., Barreiro E., Zin W. (2003). Therapeutic potential of a new phosphodiesterase inhibitor in acute lung injury. Eur. Respir. J..

[177] Świerczek A., Pociecha K., Ślusarczyk M., Chłoń-Rzepa G., Baś S., Mlynarski J., Więckowski K., Zadrożna M., Nowak B., Wyska E. (2020). Comparative Assessment of the New PDE7 Inhibitor-GRMS-55 and Lisofylline in Animal Models of Im-mune-Related Disorders: A PK/PD Modeling Approach. Pharm. Res..

[178] Lakshminrusimha S., Mathew B., Leach C.L. (2016). Pharmacologic strategies in neonatal pulmonary hypertension other than nitric oxide. Semin. Perinatol..

[179] Barton P., Garcia J., Kouatli A., Kitchen L., Zorka A., Lindsay C., Lawless S., Giroir B. (1996). Hemodynamic effects of i.v. mil-rinone lactate in pediatric patients with septic shock. A prospective, double-blinded, randomized, placebo-controlled, inter-ventional study. Chest.

[180] Wang Z., Wu Q., Nie X., Guo J., Yang C. (2015). Combination Therapy with Milrinone and Esmolol for Heart Protection in Patients with Severe Sepsis: A Prospective, Randomized Trial. Clin. Drug Investig..

[181] Kelly L.E., Ohlsson A., Shah P.S. (2017). Sildenafil for pulmonary hypertension in neonates. Cochrane Database Syst. Rev..

[182] Martinho S., Adão R., Leite-Moreira A.F., Brás-Silva C. (2020). Persistent Pulmonary Hypertension of the Newborn: Pathophysio-logical Mechanisms and Novel Therapeutic Approaches. Front. Pediatr..

[183] Ziegler J.W., Ivy D.D., Wiggins J.W., Kinsella J.P., Clarke W.R., Abman S.H. (1998). Effects of Dipyridamole and Inhaled Nitric Oxide in Pediatric Patients with Pulmonary Hypertension. Am. J. Respir. Crit. Care Med..

[184] Sulica R., Dinh H.V., Dunsky K., Fuster V., Poon M. (2005). The Acute Hemodynamic Effect of IV Nitroglycerin and Dipyridamole in Patients With Pulmonary Arterial Hypertension: Comparison With IV Epoprostenol. Congest. Hear. Fail..

[185] Sabri M.R., Beheshtian E. (2014). Comparison of the Therapeutic and Side Effects of Tadalafil and Sildenafil in Children and Adolescents with Pulmonary Arterial Hypertension. Pediatr. Cardiol..

[186] Bhogal S., Khraisha O., Al Madani M., Treece J., Baumrucker S.J., Paul T.K. (2019). Sildenafil for Pulmonary Arterial Hypertension. Am. J. Ther..

[187] Cornet A.D., Hofstra J.J., Swart E.L., Girbes A.R.J., Juffermans N.P. (2010). Sildenafil attenuates pulmonary arterial pressure but does not improve oxygenation during ARDS. Intensiv. Care Med..

[188] Vestal R.E., Eiriksson C.E., Musser B., Ozaki L.K., Halter J.B. (1983). Effect of intravenous aminophylline on plasma levels of catecholamines and related cardiovascular and metabolic responses in man. Circulation.

[189] Mokra D., Tonhajzerova I., Mokry J., Petrášková M., Hutko M., Calkovska A. (2012). Cardiovascular Side Effects of Aminophylline in Meconium-Induced Acute Lung Injury. Adv. Exp. Med. Biol..

[190] Mokra D., Tonhajzerova I., Pistekova H., Visnovcova Z., Mokry J., Drgova A., Repcakova M., Calkovska A. (2013). Short-term cardiovascular effects of selective phosphodiesterase 3 inhibitor olprinone versus non-selective phosphodiesterase inhibitor aminophylline in a meconium-induced acute lung injury. J. Physiol. Pharmacol. Off. J. Pol. Physiol. Soc..

[191] Currie G.P., Butler C.A., Anderson W.J., Skinner C. (2008). Phosphodiesterase 4 inhibitors in chronic obstructive pulmonary disease: A new approach to oral treatment. Br. J. Clin. Pharmacol..

[192] Castro A., Jerez M.J., Gil C., Martinez A. (2005). Cyclic nucleotide phosphodiesterases and their role in immunomodulatory re-sponses: Advances in the development of specific phosphodiesterase inhibitors. Med. Res. Rev..

[193] Smith W.B., McCaslin I.R., Gokce A., Mandava S.H., Trost L., Hellstrom W.J. (2013). PDE5 inhibitors: Considerations for preference and long-term adherence. Int. J. Clin. Pr..

[194] Giorgi M., Cardarelli S., Ragusa F., Saliola M., Biagioni S., Poiana G., Naro F., Massimi M. (2020). Phosphodiesterase Inhibitors: Could They Be Beneficial for the Treatment of COVID-19?. Int. J. Mol. Sci..

[195] Kuba K., Imai Y., Rao S., Gao H., Guo F., Guan B., Huan Y., Yang P., Zhang Y., Deng W. (2005). A crucial role of angiotensin converting enzyme 2 (ACE2) in SARS coronavirus–induced lung injury. Nat. Med..

[196] Imai Y., Kuba K., Penninger J.M. (2007). Angiotensin-converting enzyme 2 in acute respiratory distress syndrome. Cell. Mol. Life Sci..

[197] Hamming I., Timens W., Bulthuis M.L.C., Lely A.T., Navis G.J., Van Goor H. (2004). Tissue distribution of ACE2 protein, the functional receptor for SARS coronavirus. A first step in understanding SARS pathogenesis. J. Pathol..

[198] Jia H. (2016). Pulmonary Angiotensin-Converting Enzyme 2 (ACE2) and Inflammatory Lung Disease. Shock.

[199] El Tabaa M.M. (2020). New putative insights into neprilysin (NEP)-dependent pharmacotherapeutic role of roflumilast in treating COVID-19. Eur. J. Pharmacol..

[200] McGonagle D., Sharif K., O’Regan A., Bridgewood C. (2020). The Role of Cytokines including Interleukin-6 in COVID-19 induced Pneumonia and Macrophage Activation Syndrome-Like Disease. Autoimmun. Rev..

[201] Klok F.A., Kruip M.J.H.A., van der Meer N.J.M., Arbous M.S., Gommers D.A.M.P.J., Kant K.M., Kaptein F.H.J., van Paassen J., Stals M.A.M., Huisman M.V. (2020). Incidence of thrombotic complications in critically ill ICU patients with COVID-19. Thromb. Res..

[202] Belen-Apak F.B., Sarıalioğlu F. (2020). Pulmonary intravascular coagulation in COVID-19: Possible pathogenesis and recommenda-tions on anticoagulant/thrombolytic therapy. J. Thromb. Thrombolysis.

[203] Chiang C.-C., Korinek M., Cheng W.-J., Hwang T.-L. (2020). Targeting Neutrophils to Treat Acute Respiratory Distress Syndrome in Coronavirus Disease. Front. Pharmacol..

[204] A Insel P., Murray F., Yokoyama U., Romano S., Yun H., Brown L., Snead A., Lu D., Aroonsakool N. (2012). cAMP and Epac in the regulation of tissue fibrosis. Br. J. Pharmacol..

[205] Wermuth P.J., Li Z., Mendoza F.A., Jimenez S.A. (2016). Stimulation of Transforming Growth Factor-β1-Induced Endotheli-al-To-Mesenchymal Transition and Tissue Fibrosis by Endothelin-1 (ET-1): A Novel Profibrotic Effect of ET-1. PLoS ONE.

[206] Maldonado V., Hernandez-Ramírez C., Oliva-Pérez E.A., Sánchez-Martínez C.O., Pimentel-González J.F., Molina-Sánchez J.R., Jiménez-Villalba Y.Z., Chávez-Alderete J., Loza-Mejía M.A. (2021). Pentoxifylline decreases serum LDH levels and increases lymphocyte count in COVID-19 patients: Results from an external pilot study. Int. Immunopharmacol..

[207] A Matthay M., Aldrich J.M., E Gotts J. (2020). Treatment for severe acute respiratory distress syndrome from COVID-19. Lancet Respir. Med..

[208] Maldonado V., Loza-Mejía M.A., Chávez-Alderete J. (2020). Repositioning of pentoxifylline as an immunomodulator and regulator of the renin-angiotensin system in the treatment of COVID-19. Med. Hypotheses.

[209] Effendi W.I., Nagano T., Kobayashi K., Nishimura Y. (2020). Focusing on Adenosine Receptors as a Potential Targeted Therapy in Human Diseases. Cells.

[210] Schönharting M.M., Schade U.F. (1989). The effect of pentoxifylline in septic shock--New pharmacologic aspects of an established drug. J. Med..

[211] Lee J.-G., Shim S., Kim M.-J., Myung J.K., Jang W.-S., Bae C.-H., Lee S.-J., Kim K.M., Jin Y.-W., Lee S.-S. (2017). Pentoxifylline Regulates Plasminogen Activator Inhibitor-1 Expression and Protein Kinase A Phosphorylation in Radiation-Induced Lung Fibrosis. BioMed Res. Int..

[212] Raetsch C., Jia J.D., Boigk G., Bauer M., Hahn E.G., Riecken E.-O., Schuppan D. (2002). Pentoxifylline downregulates profibrogenic cytokines and procollagen I expression in rat secondary biliary fibrosis. Gut.

[213] Hendry B.M., Stafford N., Arnold A.D., Sangwaiya A., Manglam V., Rosen S.D., Arnold J. (2020). Hypothesis: Pentoxifylline is a potential cytokine modulator therapeutic in COVID-19 patients. Pharmacol. Res. Perspect..

[214] Ali R.A., Gandhi A.A., Meng H., Yalavarthi S., Vreede A.P., Estes S.K., Palmer O.R., Bockenstedt P.L., Pinsky D.J., Greve J.M. (2019). Adenosine receptor agonism protects against NETosis and thrombosis in antiphos-pholipid syndrome. Nat. Commun..

[215] Liu X., Li Z., Liu S., Sun J., Chen Z., Jiang M., Zhang Q., Wei Y., Wang X., Huang Y.-Y. (2020). Potential therapeutic effects of dipyridamole in the severely ill patients with COVID-19. Acta Pharm. Sin. B.

[216] Motta N.A.V., Autran L.J., Brazão S.C., Lopes R.D.O., Scaramello C.B.V., Lima G.F., De Brito F.C.F. (2021). Could cilostazol be beneficial in COVID-19 treatment? Thinking about phosphodiesterase-3 as a therapeutic target. Int. Immunopharmacol..

[217] Bridgewood C., Damiani G., Sharif K., Watad A., Bragazzi N.L., Quartuccio L., Savic S., McGonagle D. (2020). Rationale for Evaluating PDE4 Inhibition for Mitigating against Severe Inflammation in COVID-19 Pneumonia and Beyond. Isr. Med Assoc. J. IMAJ.

[218] Totani L., Amore C., Di Santo A., Dell’Elba G., Piccoli A., Martelli N., Tenor H., Beume R., Evangelista V. (2016). Roflumilast inhibits leukocyte-platelet interactions and prevents the prothrombotic functions of polymorphonuclear leukocytes and mon-ocytes. J. Thromb. Haemost..

[219] Muo I.M., Macdonald S.D., Madan R., Park S.-J., Gharib A.M., Martinez P.E., Walter M.F., Yang S.B., Rodante J.A., Courville A.B. (2019). Early effects of roflumilast on insulin sensitivity in adults with prediabetes and overweight/obesity involve age-associated fat mass loss-results of an exploratory study. Diabetes, Metab. Syndr. Obes. Targets Ther..

[220] White W.B., Cooke G.E., Kowey P.R., Calverley P.M.A., Bredenbröker D., Goehring U.-M., Zhu H., Lakkis H., Mosberg H., Rowe P. (2013). Cardiovascular Safety in Patients Receiving Roflumilast for the Treatment of COPD. Chest.

[221] Cazzola M., Calzetta L., Rogliani P., Matera M.G. (2019). Ensifentrine (RPL554): An investigational PDE3/4 inhibitor for the treat-ment of COPD. Expert. Opin. Investig. Drugs.

[222] Mokry J., Giembycz M., Mokra D. (2021). Editorial: Phosphodiesterases as Drug Targets in Airway and Inflammatory Diseases. Front Pharmacol.

